# MiFish, a set of universal PCR primers for metabarcoding environmental DNA from fishes: detection of more than 230 subtropical marine species

**DOI:** 10.1098/rsos.150088

**Published:** 2015-07-22

**Authors:** M. Miya, Y. Sato, T. Fukunaga, T. Sado, J. Y. Poulsen, K. Sato, T. Minamoto, S. Yamamoto, H. Yamanaka, H. Araki, M. Kondoh, W. Iwasaki

**Affiliations:** 1 Department of Zoology, Natural History Museum and Institute, Chiba 260-8682, Japan; 2 CREST, Japan Science and Technology Agency, Saitama 332-0012, Japan; 3 Tohoku Medical Megabank Organization, Tohoku University, Miyagi 980-8573, Japan; 4 Department of Computational Biology, The University of Tokyo, Chiba 277-8568, Japan; 5 Fish Section, Australian Museum, Sydney, New South Wales 2010, Australia; 6 Okinawa Churashima Research Center, Okinawa 905-0206, Japan; 7 Graduate School of Human Development and Environment, Kobe University, Hyogo 657-8501, Japan; 8 Faculty of Science and Technology, Ryukoku University, Shiga 520-2194, Japan; 9 Research Faculty of Agriculture, Hokkaido University, Hokkaido 060-8589, Japan; 10 Department of Biological Sciences, The University of Tokyo, Tokyo 133-0032, Japan

**Keywords:** metabarcoding, MiSeq, environmental DNA, mitogenome, resource management, community ecology

## Abstract

We developed a set of universal PCR primers (MiFish-U/E) for metabarcoding environmental DNA (eDNA) from fishes. Primers were designed using aligned whole mitochondrial genome (mitogenome) sequences from 880 species, supplemented by partial mitogenome sequences from 160 elasmobranchs (sharks and rays). The primers target a hypervariable region of the 12S rRNA gene (163–185 bp), which contains sufficient information to identify fishes to taxonomic family, genus and species except for some closely related congeners. To test versatility of the primers across a diverse range of fishes, we sampled eDNA from four tanks in the Okinawa Churaumi Aquarium with known species compositions, prepared dual-indexed libraries and performed paired-end sequencing of the region using high-throughput next-generation sequencing technologies. Out of the 180 marine fish species contained in the four tanks with reference sequences in a custom database, we detected 168 species (93.3%) distributed across 59 families and 123 genera. These fishes are not only taxonomically diverse, ranging from sharks and rays to higher teleosts, but are also greatly varied in their ecology, including both pelagic and benthic species living in shallow coastal to deep waters. We also sampled natural seawaters around coral reefs near the aquarium and detected 93 fish species using this approach. Of the 93 species, 64 were not detected in the four aquarium tanks, rendering the total number of species detected to 232 (from 70 families and 152 genera). The metabarcoding approach presented here is non-invasive, more efficient, more cost-effective and more sensitive than the traditional survey methods. It has the potential to serve as an alternative (or complementary) tool for biodiversity monitoring that revolutionizes natural resource management and ecological studies of fish communities on larger spatial and temporal scales.

## 1. Introduction

Environmental DNA (eDNA) in aquatic environments refers to genetic material found in the water column. In the case of multicellular organisms, eDNA originates from various sources, such as metabolic waste, damaged tissue or sloughed skin cells [1]. Ficetola *et al.* [2] was the first study demonstrating the use of eDNA for detecting an aquatic vertebrate species (invasive American bullfrog) from controlled environments and natural wetland, published in 2008. Subsequently, eDNA from fishes has been detected from various aquatic environments, including ponds [3–5], streams [6], rivers [7–10] and seawater [11,12]. Such ubiquitous presence of eDNA from fishes in the water column has led to the increasing use of this technique as a tool for detections of invasive [3,7–9], rare or threatened species [5,6], investigations of local fauna [10,13], or in a larger mesocosm [12] with known species composition. These pioneering studies have shown the use of eDNA to be appropriate as a non-invasive genetic monitoring tool in various fields of fish biology.

For monitoring the occurrence of a single or few fish species, short species-specific eDNA fragments (72–312 bp) have been used [3,5–9], with earlier studies detecting those species based on the presence/absence of PCR products by visually inspecting the products on an agarose gel stained with ethidium bromide [7–9]. More recently, quantitative PCR (qPCR) using probe-based chemistries has been employed for the detection of target species [3–6] owing to the method's sensitivity, specificity and potential to quantify the target DNA [6]. For example, Takahara *et al.* [4] estimated the biomass of common carp (*Cyprinus carpio*) in a natural freshwater lagoon, using the qPCR approach (real-time PCR), based on the positive relationships between eDNA concentrations and biomass in aquaria and experimental ponds.

For monitoring fish assemblages with broader taxonomic scopes, Minamoto *et al.* [10] designed degenerate PCR primers to amplify a short fragment of the mitochondrial cyt *b* gene (285 bp) with reference to those sequences from the local freshwater fish fauna. Based on PCR amplification of the fragment and subsequent subcloning and sequencing of the product, they successfully detected multiple species in eDNA from the controlled aquaria (one to five spp.) and three stations in the Yura River, central Japan (two to four spp.) [10]. Thomsen *et al.* [11] developed two generic and four species-specific PCR primer sets for amplifying short fragments of the cyt *b* gene (32–51 bp), in order to detect marine fish species from three sampling sites at a coastal zone in Denmark. Using a next-generation sequencing (NGS) platform (Roche 454 GS FLX), they detected 15 species in the amplicons, including both important commercial fishes as well as some species rarely recorded by conventional monitoring methods [11]. More recently, Kelly *et al.* [12] attempted to estimate the fish fauna in a large tank at the Monterey Bay Aquarium with known species composition by sequencing PCR amplicons from eDNA using an NGS platform (Illumina MiSeq). They used a set of published universal PCR primers to amplify a 106 bp fragment of the mitochondrial 12S rRNA gene [14] for metabarcoding fish species in the tank. Although they detected seven of the eight species of bony fishes present, they were able to identify those species only to taxonomic family or genus owing to the limited sequence variability within the amplicons. In addition, they failed to detect all three elasmobranchs (sharks and rays) contained in the tank [12].

These earlier studies on eDNA metabarcoding (high-throughput multispecies identification using degraded DNA extracted from an environmental sample [15]) have shown both potential and limitations. They are non-invasive and are demonstrably more efficient and cost-effective than the traditional monitoring methods, such as visual surveys, trawls and seines [11,12]. The former two studies [10,11], however, required development of PCR primers specifically designed with reference to DNA sequences from the known local fish fauna and those primers are of limited uses in future studies with little prior knowledge on the faunal composition. The latter study [12] employed PCR primers that have been developed using the computer software ‘ecoPrimers’ [14] and that are supposedly universal among vertebrates. Despite the use of universal primers, the successful detection in the aquarium tank was dependent on the taxonomic groups (e.g. no detection for ocean sunfish and all elasmobranchs), and the amplified products, if any, exhibited little sequence variability to correctly assign fish species in the same family or genus [12].

The primary objective of this study was to circumvent these problems associated with PCR primers. To achieve this goal, we: (i) developed universal primers for fish eDNA that amplify a short fragment (less than 200 bp) containing sufficient sequence variation to correctly assign fish species; (ii) tested versatility of the primers across a taxonomically and ecologically diverse range of fishes using eDNA from aquarium tanks with known species compositions; and (iii) preliminarily examined the use of the primers for detecting eDNA from fishes inhabiting natural seawater environments with unknown species composition and abundances in an open ecosystem.

The development of the universal primers (MiFish-U/E) was based on the aligned whole mitochondrial genome (mitogenome) sequences from 880 fish species, which was supplemented by partial mitogenome sequences from 160 elasmobranchs. The primers are targeted to amplify a hypervariable region of the 12S rRNA gene (163–185 bp), which contains sufficient information to unambiguously identify fishes we tested to taxonomic family, genus and species, with one exception (closely related congeners of *Thunnus*). We tested the versatility of those PCR primers using eDNA from four tanks in the Okinawa Churaumi Aquarium and from natural seawaters near the aquarium in the subtropical western North Pacific. Using a high-throughput Illumina MiSeq platform, we detected eDNA from 232 fish species from those seawaters, which are taxonomically diverse and are distributed across 70 families and 152 genera. In addition to eDNA, this metabarcoding approach is applicable to bulk samples (total DNA), such as those from net collections containing a diverse range of fish eggs, larvae, juveniles or damaged specimens with few diagnostic characters present for species identification.

## 2. Material and methods

### 2.1 Primer development

#### 2.1.1 Selection of genetic marker

Mitochondrial DNA (mtDNA) was chosen as the genetic marker because copy number of mtDNA is greater than that of nuclear DNA per cell, and detection rate therefore is expected to be higher in the former, even where DNA is present at a low concentration and/or is degraded [16]. In order to select a suitable region in the mitogenome for species identification based on eDNA, 1044 whole mitogenome sequences were batch downloaded from the database MitoFish v. 2.80 [17] in a FASTA format as of 20 April 2013. After removing problematic sequences involving large-scale gene rearrangements [18], the remaining 880 sequences (electronic supplementary material, table S1) were subjected to multiple alignment using MAFFT v. 6.956 [19] with a default set of parameters. The aligned sequences were imported into Mesquite v. 2.75 [20] for visual inspection of the conservative and hypervariable regions. The search for a short hypervariable region (up to 200 bp for paired-end sequencing using the Illumina MiSeq) flanked by two conservative regions (*ca* 20–30 bp) across 880 species was performed on the entire set of aligned mitogenomes. The conservative and hypervariable regions were highlighted by a ‘Select’ function in Mesquite (a submenu ‘Variable among taxa’ in ‘Select Characters’) [20].

#### 2.1.2 Primer design

To facilitate primer design based on comparisons of diverse sequences from 880 fish species, a base composition for a selected position in the conservative region was shown using a ‘Show Selection Summary Strip’ function in Mesquite [20]. The base compositions in selected characters were manually recorded in a spreadsheet for the primer design. In the primer design process, we considered a number of technical tips that enhance the primer annealing to the template without the uses of degenerate bases [21]: primers include some G/C at the 3′-ends to strengthen primer–template annealing at this position, but a string of either Gs or Cs at the 3′-end should be avoided; considering the unconventional base pairing in the T/G bond, the designed primers use G rather than A when the template is variably C or T, and T rather than C when the template is A or G; G/C contents of the primers fall between 40 and 60% with an almost identical melting temperature (*T*_*m*_). *T*_*m*_ was calculated using a nearest-neighbour thermodynamic model implemented in OligoCalc [22].

The first universal primers for eDNA were designed on the 12S rRNA gene (for details, see Results and Discussion) and were named MiFish-U-F/R (with overhang adapter sequences for library preparation; U, F and R represent universal, forward and reverse, respectively). In addition, we had to design MiFish-E-F/R to accommodate sequence variations in the priming sites of elasmobranchs (E), with the primer designs based on newly assembled partial mitogenome sequences from 160 species (electronic supplementary material, table S2). For more accurate species assignments within closely related congeners, we also designed genus-specific primers that amplify a different mitogenomic gene (ND5) with significant variations across constituent species (e.g. MiFish-tuna).

#### 2.1.3 Primer testing with extracted DNA

In order to test whether these newly designed PCR primers were universal or not, we first tested MiFish-U-F/R (no adapter sequences) using extracted DNA from 96 species representing all the four major lineages of fishes (Agnatha, Chondrichthyes, Actinopterygii and Sarcopterygii) placed in 47 orders and 96 different families (table 1). Double-stranded DNA concentrations from those fishes were measured with a NanoDrop Lite spectrophotometer (Thermo Fisher Scientific, Wilmington, DE, USA) and the extracted DNA was diluted to 15 ng μl^−1^ using Milli-Q water. PCR was carried out with 30 cycles of a 15 μl reaction volume containing 8.3 μl sterile distilled H_2_O, 1.5 μl 10×PCR buffer (Takara, Otsu, Japan), 1.2 μl dNTPs (4 mM), 1.5 μl of each primer (5 μM), 0.07 μl *Taq* polymerase (Z *Taq*; Takara) and 1.0 μl template. The thermal cycle profile after an initial 2 min denaturation at 94°C was as follows: denaturation at 98°C for 5 s; annealing at 50°C for 10 s; and extension at 72°C for 10 s with the final extension at the same temperature for 5 min.

**Table 1.** A list of fish species for testing MiFish-U primers (without adapter sequences) using extracted DNA diluted to 15 ng μl^−1^, subsequently sequenced with a Sanger method.

higher classification	family	species	common name	accession no.
Class Myxini				
Order Myxiniformes	Myxinidae	*Eptatretus burgeri*	inshore hagfish	AB938082
Class Chondrichthyes				
Subclass Holocephali				
Order Chimaeriformes	Chimaeridae	*Chimaera phantasma*	silver chimaera	AB938084
Subclass Elasmobranchii				
Subdivision Selachii				
Order Carcharhiniformes	Triakidae	*Mustelus griseus*	spotless smooth-hound	AB938092
Order Squaliformes	Squalidae	*Cirrhigaleus barbifer*	mandarin dogfish	AB938108
Order Pristiophoriformes	Pristiophoridae	*Pristiophorus japonicus*	Japanese sawshark	AB938111
Subdivision Batoidea				
Order Torpediniformes	Torpedinidae	*Torpedo tokionis*	trapezoid torpedo	AB938112
Order Rajiformes	Rhinobatidae	*Rhinobatos schlegelii*	brown guitarfish	AB974648
Class Actinopterygii				
Subclass Cladistia				
Order Polypteriformes	Polypteridae	*Polypterus senegalus*	grey bichir	AB969828
Subclass Chondrostei				
Order Acipenseriformes	Acipenseridae	*Huso dauricus*	kaluga	AB969829
Subclass Neopterygii				
Order Lepisosteiformes	Lepisosteidae	*Atractosteus spatula*	alligator gar	AB969830
Division Teleostei				
Order Osteoglossiformes	Osteoglossidae	*Osteoglossum bicirrhosum*	arowana	AB969831
Order Elopiformes	Megalopidae	*Megalops cyprinoides*	Indo-Pacific tarpon	AB969832
Order Albuliformes				
Suborder Notacanthoidei	Notacanthidae	*Notacanthus chemnitzi*	spiny eel	AB969833
Order Anguilliformes				
Suborder Anguilloidei	Anguillidae	*Anguilla marmorata*	giant mottled eel	AB969834
	Muraenidae	*Muraena pardalis*	leopard moray eel	AB969835
Order Clupeiformes				
Suborder Denticipitoidei	Denticipitidae	*Denticeps clupeoides*	denticle herring	AB969840
Suborder Clupeoidei	Clupeidae	*Sardinella lemuru*	Bali sardinella	AB969841
Order Gonorynchiformes				
Suborder Chanoidei	Chanidae	*Chanos chanos*	milkfish	AB969842
Order Cypriniformes	Cyprinidae	*Gnathopogon elongatus elongatus*	Tamoroko gudgeon	AB969843
Order Characiformes				
Suborder Characoidei	Characidae	*Exodon paradoxus*	bucktooth tetra	AB969844
Order Siluriformes	Bagridae	*Pseudobagrus virgatus*	Gibachi bagrid catfish	AB969845
Order Gyrnnotiformes	Gymnotidae	*Gymnotus carapo*	banded knifefish	AB969846
Order Argentiniformes				
Suborder Argentinoidei	Argentinidae	*Glossanodon semifasciatus*	deep-sea smelt	LC020812
Order Osmeriformes	Osmeridae	*Hypomesus japonicus*	Japanese smelt	AB969847
Order Salmoniformes	Salmonidae	*Oncorhynchus masou*subsp.	masu salmon	AB969848
Order Esociformes	Esocidae	*Esox americanus*	redfin pickerel	AB969849
Order Stomiiformes				
Suborder Gonostomatoidei	Gonostomatidae	*Sigmops longipinnis*	elongated bristlemouth fish	AB969850
Order Ateleopodiformes	Ateleopodidae	*Ateleopus japonicus*	Pacific jellynose fish	AB969853
Order Aulopiformes				
Suborder Synodontoidei	Synodontidae	*Saurida macrolepis*	Ma-eso lizardfish	AB938170
Order Myctophiformes	Myctophidae	*Diaphus watasei*	Watases lanternfish	AB938172
Order Lampriformes	Trachipteridae	*Trachipterus ishikawae*	slender ribbonfish	AB938162
Order Polymixiiformes	Polymixiidae	*Polymixia longispina*	silver eye	LC020813
Order Percopsiformes	Percopsidae	*Percopsis transmontana*	sand roller	AB969861
Order Gadiformes	Macrouridae	*Trachyrincus murrayi*	roughnose grenadier	AB969865
	Gadidae	*Theragra chalcogramma*	Alaska pollock	AB969867
Order Ophidiiformes				
Suborder Ophidioidei	Carapidae	*Carapus bermudensis*	pearlfish	AB969871
Suborder Bythitioidei	Bythitidae	*Cataetyx rubrirostris*	rubynose brotula	AB969872
Order Lophiiformes				
Suborder Ogcocephalioidei	Ogcocephalidae	*Chaunax abei*	Japanese sea toad	AB969874
	Melanocetidae	*Melanocetus murrayi*	Murray's abyssal anglerfish	LC020814
Order Mugiliformes	Mugilidae	*Chelon labrosus*	thicklip grey mullet	AB969954
Order Atheriniformes	Atherinidae	*Hypoatherina tsurugae*	Gin-iso-iwashi silverside	AB974688
Order Beloniformes	Adrianichthyidae	*Oryzias latipes*	Japanese rice fish	AB969878
	Belonidae	*Cypselurus pinnatibarbatus japonicus*	Bennett's flyingfish	AB969879
Order Cyprinodontiformes	Poeciliidae	*Xiphophorus maculatus*	southern platyfish	AP005982
Order Stephanoberyciformes	Melamphaidae	*Scopelogadus*sp.	bigscale	AB969880
Order Beryciformes				
Suborder Berycoidei	Berycidae	*Beryx decadactylus*	alfonsino	AB969882
Order Zeiformes				
Suborder Zeioidei	Zeniontidae	*Zenion japonicum*	Japanese dory	AB969885
Order Gasterosteiformes				
Suborder Gasterosteoidei	Aulorhynchidae	*Aulichthys japonicus*	tubenose	AB969886
Order Synbranchiformes				
Suborder Synbranchoidei	Synbranchidae	*Synbranchus marmoratus*	marbled swamp eel	AB972265
Order Scorpaeniformes				
Suborder Scorpaenoidei	Scorpaenidae	*Sebastes schlegelii*	Korean rockfish	AB969888
	Tetrarogidae	*Paracentropogon rubripinnis*	Haokoze wasp fish	AB938167
	Peristediidae	*Scalicus serrulatus*	Kihoubou armored searobin	AB969898
Suborder Platycephaloidei	Platycephalidae	*Platycephalus*sp.	Magochi flathead	AB969904
Suborder Cottoidei	Cottidae	*Pseudoblennius percoides*	sunrise	AB969909
		*Hemitripterus villosus*	shaggy sculpin	AB938165
	Cyclopteridae	*Eumicrotremus pacificus*	Fusen-uo lampfish	AB974680
	Liparidae	*Careproctus rastrinus*	salmon snailfish	AB974681
Order Perciformes				
Suborder Percoidei	Moronidae	*Lateolabrax latus*	blackfin seabass	AB938173
	Serranidae	*Epinephelus akaara*	Hong Kong grouper	AB974679
	Opistognathidae	*Opistognathus punctatus*	finespotted jawfish	AB972248
	Priacanthidae	*Pristigenys niphonia*	Japanese bigeye	AB972242
	Apogonidae	*Siphamia majimai*	striped siphonfish	LC020815
	Carangidae	*Selar crumenophthalmus*	bigeye scad	AB938143
	Bramidae	*Taractichthys steindachneri*	sickle pomfret	AB938175
	Lutjanidae	*Lutjanus kasmira*	common bluestripe snapper	AB938146
	Lobotidae	*Lobotes surinamensis*	tripletail	AB972214
	Haemulidae	*Parapristipoma trilineatum*	chicken grunt	AB972213
	Nemipteridae	*Nemipterus bathybius*	yellowbelly threadfin bream	AB972211
	Lethrinidae	*Gymnocranius griseus*	grey large-eye bream	AB938151
	Sparidae	*Acanthopagrus schlegelii*	blackhead seabream	AB972186
	Sciaenidae	*Boesemania microlepis*	boeseman croaker	AB972206
	Mullidae	*Parupeneus ciliatus*	whitesaddle goatfish	AB972204
	Chaetodontidae	*Chaetodon auripes*	oriental butterflyfish	AB972196
	Pentacerotidae	*Evistias acutirostris*	striped boarfish	AB972192
	Terapontidae	*Terapon jarbua*	Jarbua terapon	AB972191
	Oplegnathidae	*Oplegnathus fasciatus*	barred knifejaw	AB972189
	Cheilodactylidae	*Goniistius zonatus*	spottedtail morwong	AB938161
Suborder Labroidei	Cichlidae	*Thorichthys meeki*	firemouth cichlid	AB972187
	Embiotocidae	*Ditrema viride*	Umi-tanago surfperch	AB969918
	Labridae	*Cheilio inermis*	cigar wrasse	AB972174
Suborder Zoarcoidei	Stichaeidae	*Stichaeus grigorjewi*	Nagazuka prickleback	AB972145
Suborder Notothenioidei	Eleginopidae	*Eleginops maclovinus*	Patagonian blennie	AB969976
Suborder Trachinoidei	Arnmodytidae	*Ammodytes personatus*	Pacific sandlance	AB969933
	Uranoscopidae	*Xenocephalus elongatus*	bluespotted stargazer	AB969930
Suborder Blennioidei	Blenniidae	*Entomacrodus striatus*	reef margin blenny	AB969913
Suborder Icosteoidei	Icosteidae	*Icosteus aenigmaticus*	ragfish	AB972142
Suborder Gobioidei	Gobiidae	*Schismatogobius roxasi*	Eso-haze goby	AB972140
Suborder Acanthuroidei	Scatophagidae	*Scatophagus argus*	spotted scat	AB969929
Suborder Scombroidei	Gempylidae	*Lepidocybium flavobrunneum*	escolar	AB972115
	Scombridae	*Gymnosarda unicolor*	dogtooth tuna	AB972114
Suborder Stromateoidei	Stromateidae	*Pampus punctatissimus*	Managatsuo butterfish	AB972108
Suborder Channoidei	Channidae	*Channa argus*	snakehead	AB972107
Order Pleuronectiformes				
Suborder Pleuronectoidei	Paralichthyidae	*Paralichthys olivaceus*	bastard halibut	AB972104
	Cynoglossidae	*Paraplagusia japonica*	black cow-tongue	AB972088
Order Tetraodontiformes				
Suborder Balistoidei	Monacanthidae	*Chaetodermis penicilligera*	prickly leatherjacket	AB972083
Suborder Tetraodontoidei	Tetraodontidae	*Arothron hispidus*	white-spotted puffer	AB972076

Double-stranded PCR products were purified using Exo SAP-IT (USB, Cleveland, OH, USA) to remove redundant dNTPs and oligonucleotides from primers. Direct cycle sequencing was performed with dye-labelled terminators (BigDye terminator v. 1.1; Applied Biosystems, Foster City, CA, USA) following the manufacturer's protocol and the purified PCR products were sequenced for both strands on the ABI 3130*xl* Genetic Analyzer (Life Technologies, Carlsbad, CA, USA). The DNA sequences were edited and assembled using GENETYX-MAC v. 17 (Genetyx, Tokyo, Japan) and deposited in DDBJ/EMBL/GenBank databases.

#### 2.1.4 *In silico* evaluation of interspecific variation

Interspecific differences within the amplified DNA sequences are required for accurate assignments of taxonomic categories. To computationally evaluate levels of interspecific variation in the target region (hereafter called ‘MiFish sequence’) across different taxonomic groups of fishes, 1361 whole mitogenome sequences were batch downloaded from MitoFish v. 2.89 [17] as of 3 September 2014. After removing duplicate sequences (e.g. multiple sequences from subspecies), uncertain taxonomic status (e.g. hybrids) and possible erroneous sequences (e.g. unable to annotate using MitoAnnotator [17]), the MiFish sequences were extracted from the remaining 1324 sequences using custom Ruby scripts (available from: http://dx.doi.org/10.5061/dryad.54v2q) and they were subjected to calculation of pairwise edit distances. The edit distance quantifies dissimilarity of sequences in bioinformatics [23] and is defined as the minimum number of single-nucleotide substitutions, insertions or deletions that are required to transform one sequence into the other. For comparisons, metabarcode sequences amplified by *12S-V5* primers [14] (forward: 5′-ACTGGGATTAGATACCCC-3′; and reverse: 5′-TAGAACAGGCTCCTCTAG-3′) (hereafter called ‘ecoPrimer sequences’) were also extracted from the 1324 sequences and their interspecific variation was evaluated as described for MiFish sequences. The ecoPrimer pair amplifies the same gene (mitochondrial 12S rRNA gene) as that of the MiFish-U/E primers, but the two primer pairs are designed to amplify two different regions adjacent to each other (*12S-V5-F* primer is located within MiFish-U-R primer). The ecoPrimer pair was used in a metabarcoding study of fishes by Kelly *et al.* [12] who attempted to estimate an artificial fish fauna using eDNA in the large tank at the Monterey Bay Aquarium.

### 2.2 Primer testing with environmental DNA

#### 2.2.1 Sampling sites

**Figure 1.**
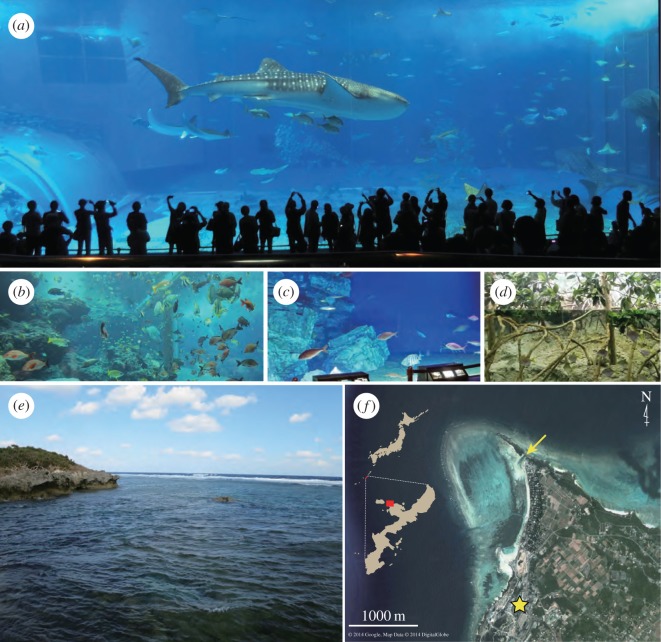
(*a*–*d*) Four tanks used for water sampling in the Okinawa Churaumi Aquarium and (*e*,*f*) a sampling site in the coral reefs near the aquarium: (*a*) Kuroshio (water volume =7500 m^3^); (*b*) tropical fish (700 m^3^); (*c*) deep-sea (230 m^3^); and (*d*) mangrove (35.6 m^3^) tanks; (*e*,*f*) sampling site in Bise (arrow; 26°42′35^′′^ N, 127°52′48^′′^ E) and the Okinawa Churaumi Aquarium (star; 26°41′39^′′^ N, 127°52′41^′′^ E).

In order to test the versatility of the newly designed primers for metabarcoding eDNA from fishes, we sampled seawater from four tanks in the Okinawa Churaumi Aquarium, Okinawa, Japan (26°41′39′′ N, 127°52′41^′′^ E; figure 1). The aquarium was chosen because of the remarkable taxonomic diversity of fishes contained in a variety of tanks that resemble surrounding environments in the subtropical western North Pacific. The four selected tanks; Kuroshio (water volume =7500 m^3^), tropical fish (700 m^3^), deep-sea (230 m^3^) and mangrove (35.6 m^3^) tanks (figure 1*a*–*d*) harbour diverse groups of fishes (*ca* 250 species) from elasmobranchs (sharks and rays) to higher teleosts that vary greatly in their ecology, including both pelagic and benthic species living in shallow coastal to deep waters. In addition to these four aquarium tanks, we also sampled seawaters from coral reefs nearby the aquarium (26°42′35^′′^ N, 127°52′48^′′^ E; figure 1*e*,*f*) to preliminarily examine the use of the primers for metabarcoding eDNA from natural environments with unknown fish composition and abundances in an open ecosystem.

#### 2.2.2 Water sampling and DNA extraction

All sampling and filtering equipment was exposed to a 10% bleach solution for at least 30 min before use. For water samplings in the aquarium, approximately 10 l of seawater was collected from the surface using multiple casts of an 8 l polyethylene bucket fastened to a 10 m rope. The bucket was thoroughly prewashed with tank water. The sampling was conducted between 10.00 and 13.00 before daily feeding on two consecutive days (2 and 3 June 2014). The sampled water was stored in a valve-equipped 10 l book bottle and immediately brought to the laboratory before subsequent filtering. For water samples from the coral reefs near the aquarium, 10 l of seawater was collected in a similar manner on 4 June and 7 November 2014.

One to three 2 l lots of seawater from the 10 l samples were vacuum-filtered onto 47 mm diameter glass-fibre filters (nominal pore size, 0.7 μm; Whatman, Maidstone, UK). Each filter was wrapped in commercial aluminium foil and stored in −20°C before eDNA extraction. Two litres of Milli-Q water was used as the negative control and treated identically to the eDNA samples, to monitor contamination during the filtering and subsequent DNA extraction.

DNA was extracted from the filters using the DNeasy Blood and Tissue Kit (Qiagen, Hilden, Germany) in combination with a spin column (EZ-10; Bio Basic, Markham, Ontario, Canada). After removing the attached membrane from the spin column (EZ-10), the filter was tightly folded into a small cylindrical shape and placed in the spin column. The spin column was centrifuged at 6000*g* for 1 min to remove redundant seawater for DNA extraction. The column was then placed in a new 2 ml tube and subjected to lysis using proteinase K. Before lysis, Milli-Q water (400 μl), proteinase K (20 μl) and buffer AL (180 μl) were mixed and the mixed solution was gently pipetted onto the folded filter in the spin column. The column was then placed on a 56°C preheated aluminium heat block and incubated for 30 min. The spin columns were covered with commercial aluminium foil and a clean blanket for effective incubation at the specified temperature. After the incubation, the spin column was centrifuged at 6000*g* for 1 min to collect the DNA. In order to increase DNA yields from the filter, 300 μl of sterilized TE buffer was gently pipetted onto the folded filter and the spin column was again centrifuged at 6000*g* for 1 min. The collected DNA solution (*ca* 900 μl) was purified using the DNeasy Blood and Tissue Kit following the manufacture's protocol.

#### 2.2.3 Paired-end library preparation and MiSeq sequencing

Two to five eDNA samples from each of the four aquarium tanks (total 14 samples; figure 1*a*–*d*) and four eDNA samples from the coral reefs (figure 1*e*,*f*) were used for multiplex PCR using two universal primer pairs (MiFish-U/E). Of these 18 eDNA samples, five samples from the Kuroshio tank were additionally used for multiplex PCR using two universal plus one genus-specific primer pairs (MiFish-U/E/tuna) for correct assignments of *Thunnus* species.

Prior to library preparation, work-space and equipment were sterilized, filtered pipet tips were used and separation of pre- and post-PCR was carried out to safeguard against contamination. We also employed controls to monitor contamination including PCR blanks for each experiment.

**Figure 2.**
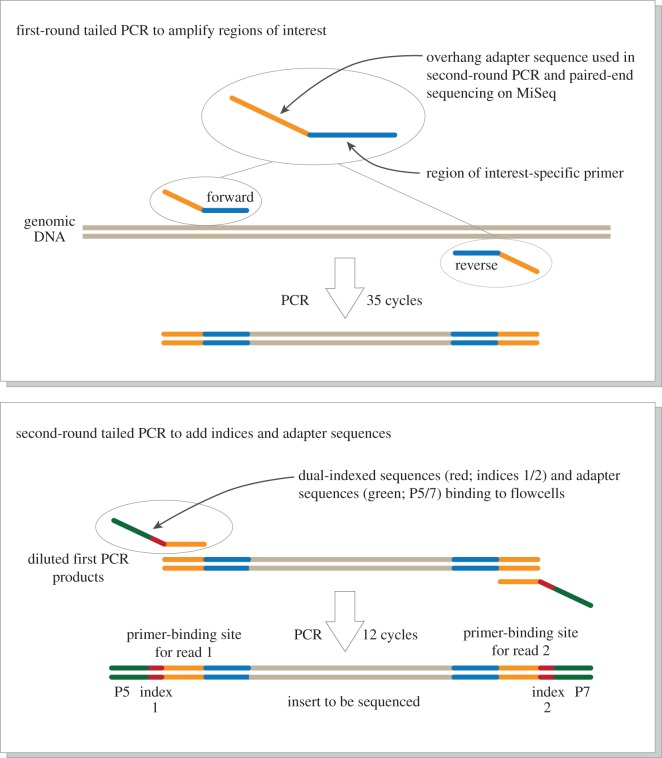
Schematic representation of the paired-end library preparation using a two-step tailed PCR. The workflow is derived from a document ‘16S metagenomic sequencing library preparation: preparing 16S ribosomal gene amplicons for the Illumina MiSeq system’ distributed by Illumina (part no. 15044223 Rev. B) and the figure was drawn with reference to a website of the Genomics and Sequencing Center at the University of Rhode Island (http://web.uri.edu/gsc/next-generation-sequencing/).

Massively parallel paired-end sequencing on the MiSeq platform (Illumina, San Diego, CA, USA) requires PCR amplicons to be flanked by: (i) primer-binding sites for sequencing; (ii) dual-index (i.e. barcode) sequences; and (iii) adapter sequences for binding to the flowcells of the MiSeq. We employed a two-step tailed PCR approach to construct the paired-end libraries (figure 2).

The first-round PCR (first PCR; figure 2) amplified the target region using primers 5′-ACACTCTTTCCCTACACGACGCTCTTCCGATCTNNNNNN + MiFish gene-specific sequences-3′ (forward) and 5′-GTGACTGGAGTTCAGACGTGTGCTCTTCCGATCTNNNNNN + MiFish gene-specific sequences-3′ (reverse). The first 33 and 34 nucleotides (nt) are partially used for primer-binding sites for sequencing and the following six random hexamers (N) are used to enhance cluster separation on the flowcells during initial base call calibrations on the MiSeq platform.

The first PCR was carried out with 35 cycles of a 12 μl reaction volume containing 6.0 μl 2×KAPA HiFi HotStart ReadyMix (including DNA polymerase, reaction buffer, dNTPs and MgCl_2_ (at a final concentration of 2.5 mM)) (KAPA Biosystems, Wilmington, MA, USA), 0.7 μl of each primer (5 μM), 2.6 μl sterile distilled H_2_O and 2.0 μl template. When the first PCR was multiplexed (simultaneous use of multiple primer pairs), the final concentration of each primer was 0.3 μM and sterile distilled H_2_O was added up to the total reaction volume of 12.0 μl. The thermal cycle profile after an initial 3 min denaturation at 95°C was as follows: denaturation at 98°C for 20 s; annealing at 65°C for 15 s; and extension at 72°C for 15 s with the final extension at the same temperature for 5 min.

The second-round PCR (second PCR; figure 2) used the first PCR products as a template and amplified the region using primers 5′-AATGATACGGCGACCACCGAGATCTACAXXXXXXXXACACTCTTTCCC TACACGACGCTCTTCCGATCT-3′ (forward) and 5′-CAAGCAGAAGACGGCATACGAGATXXXXXX XXGTGACTGGAGTTCAGACGTGTGCTCTTCCGATCT-3′ (reverse). The octo-X segments represent dual-index sequences (40 unique indices in total; A501–508, A701–712 and D501–508, D701–D712; Illumina); the 5′-end sequences are adapters that allow the final product to bind or hybridize to short oligos on the surface of the Illumina flowcell; and the 3′-end sequences are priming sites for the MiSeq sequencing.

The first PCR product was diluted 10 times using Milli-Q water and used as a template for the second PCR. The second PCR was carried out with 12 cycles of a 12 μl reaction volume containing 6.0 μl 2× KAPA HiFi HotStart ReadyMix, 0.7 μl each primer (5 μM), 3.6 μl sterile distilled H_2_O and 1.0 μl template. Different combinations of indices (chosen from A/D501–508 for forward primers and A/D701–712 for reverse primers) were used for different templates for a massively parallel sequencing using the MiSeq platform. The thermal cycle profile after an initial 3 min denaturation at 95°C was as follows: denaturation at 98°C for 20 s; annealing and extension combined at 72°C (shuttle PCR) for 15 s with the final extension at the same temperature for 5 min.

The indexed second PCR products were pooled in equal volumes and the pooled libraries (total 100 μl) were subjected to agarose gel electrophoresis using 2% L03 (Takara). A target size of the libraries (*ca* 370 bp) was excised from the gel and purified using a MinElute Gel Extraction kit (Qiagen) with an elution volume of 12 μl. The library concentration was estimated using a Qubit dsDNA HS assay kit and a Qubit fluorometer (Life Technologies). Double-stranded DNA concentration of the pooled library was adjusted to 4 nM (assuming 1 bp equals 660 g mol^−1^) using Milli-Q water and 5 μl of the 4 nM library was denatured with 5 μl of fresh 0.1 N NaOH. Including HT1 buffer (provided by the Illumina MiSeq v. 2 Reagent kit for 2×150 bp PE), the denatured library (10 μ*l*; 2 nM) was diluted to the final concentration of 12 pM for sequencing on the MiSeq platform. A 30 μ*l* of PhiX DNA spike-in control (12 pM) was added to improve data quality of low diversity samples such as single PCR amplicons used in this study.

#### 2.2.4 Data pre-processing

An overall quality of the MiSeq reads was evaluated by the programs FastQC (available from http://www.bioinformatics.babraham.ac.uk/projects/fastqc/) and SUGAR [24]. After confirming a lack of technical errors in the MiSeq sequencing, low-quality tails were trimmed from each read using DynamicTrim.pl from the SolexaQA software package [25] with a cut-off threshold set at a Phred score of 10 (=10^−1^ error rate) [26]. The tail-trimmed paired-end reads (reads 1 and 2) were assembled using the software FLASH [27] with a minimum overlap of 10 bp. The assembled reads were further filtered by custom Perl scripts in order to remove reads with either ambiguous sites (Ns) or those showing unusual lengths with reference to the expected size of the PCR amplicons (297 ± 25 bp). Finally, the software TagCleaner [28] was used to remove primer sequences with a maximum of three-base mismatches and to transform the FASTQ [29] format into FASTA.

#### 2.2.5 Taxonomic assignment

The pre-processed reads from the above custom pipeline were dereplicated using a ‘derep_fulllength’ command in UCLUST [30], with the number of identical reads added to the header line of the FASTA formatted data file. Those sequences represented by more than or equal to 10 identical reads were subjected to the downstream analyses and the remaining under-represented sequences (with less than 10 identical reads) were subjected to pairwise alignment using a ‘usearch_global’ command in UCLUST. If the latter sequences observed from less than 10 reads showed more than or equal to 99% identity with one of the former reads (one or two nucleotide differences), they were operationally considered as identical (owing to sequencing or PCR errors and/or actual nucleotide variations in the populations) and they were added to the more than or equal to 10 reads.

The processed reads were subjected to local BLASTN searches [31] against a custom-made database. The latter was generated by downloading all whole and partial fish mitogenome sequences deposited in MitoFish [17] and whole mitogenome sequences from tetrapods deposited in NCBI Organelle Genome Resources (http://www.ncbi.nlm.nih.gov/genomes/OrganelleResource.cgitaxid=32523) to cover those tetrapods occurring in aquatic environments. In addition, the custom database was supplemented by assembling new sequences in M.M.'s laboratory (electronic supplementary material, table S3). As of 4 October 2014, the database covers approximately 4230 fish species distributed across 457 families and 1827 genera. According to the latest edition of ‘Fishes of the World’ [32], fishes comprise 515 families, 1827 genera and 27 977 species with our custom-made database covering 88.7% of the families, 40.6% of the genera and 15.1% of the species.

The top BLAST hit with a sequence identity of more than or equal to 97% and *E*-value threshold of 10^−5^ was applied to species assignments of each representative sequence. We found that this cut-off value maximally recovered the species composition from each tank, while avoiding erroneous taxonomic assignment. Reliability of the species assignments were evaluated based on a ratio of total alignment length and number of mismatch bases between the query and reference sequences. For example, if a query sequence was aligned to the top BLAST hit sequence with an alignment length of 150 bp with one mismatch present, the ratio was calculated as 150/(1+1). Value one is added to the denominator to avoid zero-divisors. This ratio was calculated for the top and second BLAST hit species, and a log of odds ratio (LOD) score between these ratios was used as the comparable indicator of the species assignment. Results from the BLAST searches were automatically tabulated, with scientific names, common names, total number of the reads and representative sequences noted in an HTML format. Moreover, biological information for each detected species is available from the hyperlink in the table, such as that of FishBase (http://fishbase.sinica.edu.tw), Barcode of Life (http://www.boldsystems.org), GBIF (http://data.gbif.org), MitoFish (http://mitofish.aori.u-tokyo.ac.jp) and NCBI (http://www.ncbi.nlm.nih.gov) for quick evaluation and credibility of the bioinformatic identification.

The above bioinformatic pipeline from data pre-processing through taxonomic assignment (including Perl scripts) is available from http://dx.doi.org/10.5061/dryad.n245j and the function will be publicly available in MitoFish (http://mitofish.aori.u-tokyo.ac.jp).

## 3. Results and discussion

### 3.1 Primer development

#### 3.1.1 MiFish-U

We visually inspected the aligned sequences throughout the entire mitogenomes across the 880 species (electronic supplementary material, table S1) by highlighting variable and invariable sites using Mesquite [20]. After repeated inspections, we found a short hypervariable region (*ca* 170 bp) within the 12S rRNA gene, which was flanked by highly conservative regions (*ca* 20–30 bp) across the 880 species (table 2). Note that we were unable to find such a region within the barcoding region of the aligned COI gene sequences, which have been frequently used as the marker of choice also in fishes [33]. This observation is consistent with a recent argument against the use of the COI gene as a genetic marker for metabarcoding studies [34].

**Table 2.** Nucleotide sequences of the universal primers (MiFish-U) and base compositions in the selected 880 fish species (see electronic supplementary material, table S1). (This forward (F) and reversal (R) primer pair amplifies the mid region of the mitochondrial 12S rRNA gene with a mean length of 172 bp (163–185 bp).)

MiFish-U-F	5′-	G	T	C	G	G	T	A	A	A	A	C	T	C	G	T	G	C	C	A	G	C	-3′						
	A	20	0	1	1	0	0	786	879	879	804	0	0	0	0	0	0	0	0	880	0	0							
	C	1	733	855	0	0	6	30	0	0	17	832	3	878	0	0	0	880	880	0	0	880							
	G	858	0	0	879	880	0	0	1	0	3	0	0	0	880	0	880	0	0	0	880	0							
	T	1	147	24	0	0	874	64	0	1	56	48	877	2	0	880	0	0	0	0	0	0							

MiFish-U-R	3′-	G	T	T	T	G	A	C	C	C	T	A	A	T	C	T	A	T	G	G	G	G	T	G	A	T	A	C	-5′
	A	0	880	880	880	0	0	17	2	0	880	0	0	877	0	877	1	880	0	0	0	0	878	1	0	880	0	0	
	C	880	0	0	0	880	4	0	0	0	0	0	0	2	0	0	2	0	880	880	880	863	0	859	0	0	0	0	
	G	0	0	0	0	0	0	863	878	880	0	0	0	0	880	3	12	0	0	0	0	0	2	0	0	0	0	880	
	T	0	0	0	0	0	876	0	0	0	0	880	880	1	0	0	865	0	0	0	0	17	0	20	880	0	880	0	

The hypervariable region in the 12S rRNA gene includes multiple segments that are forming big loops in a proposed secondary structure of the molecule [35,36]. In particular, four segments of the loops were so variable in length (involving multiple insertions/deletions) that they were considered unalignable even among closely related gobioid fishes in a previous study [37]. The two highly conservative regions, on the other hand, exhibit no length variations among the 880 species and were located on the two stem regions (stem nos. 15/16 and 24/25 in [35,36]), which undergo secondary structural constraints through strong Watson–Crick base pairings [35]. Following these empirical and theoretical observations, we decided to design a new primer pair located on the two conservative regions, thereby amplifying the highly taxonomic informative hypervariable region in between.

In the initial stage of this study, we designed degenerate PCR primers to accommodate sequence variations among taxa, but found that such degenerate primers did not amplify the target eDNA when they were used with long adapter sequences in the tailed PCR (figure 2). We redesigned a new set of primers without degenerate sites (MiFish-U) using various technical methods related to construction of adequate primers (see Material and methods). The new forward (MiFish-U-F) and reverse (MiFish-U-R) primers consist of 21 and 27 bases (table 2) with G/C contents of 57% and 44% and *T*_*m*_ of 56.6°C and 56.5°C, respectively.

With the redesigned MiFish-U primers (without adapter sequences), we confirmed successful amplifications of the hypervariable regions using extracted DNA from 96 species representing all of the four major lineages of fishes (Agnatha, Chondrichthyes, Actinopterygii and Sarcopterygii) distributed across 47 orders and 96 different families (table 1). With these PCR products, we successfully determined their nucleotide sequences using the conventional Sanger sequencing method. All the sequence data are available from DDBJ/EMBL/GenBank databases with accession numbers shown in table 1.

#### 3.1.2 MiFish-E

During the preliminary experiments using eDNA from the aquarium tanks, we found that only a few assembled reads from the MiSeq sequencing represented elasmobranchs (sharks and rays). The lack of elasmobranch sequences was totally unexpected, because we included a number of elasmobranchs while designing the universal primers (13 spp.; see the electronic supplementary material, table S1) and more than 100 large-sized individuals of various elasmobranchs (mostly more than 1 m in total lengths; figure 1*a*) were present and active in the Kuroshio tank. We suspected that absence of the elasmobranch sequences resulted from PCR bias derived from primer–template mismatches. Inspection of the newly downloaded 160 elasmobranch sequences found only a few such mismatches (table 3), with significant ones being restricted to two sites near the 5′-end of the forward primer and in a single site near the 3′-end of the reverse primer. The newly designed primers for the elasmobranchs based on these mismatches were proved effective for amplification of the region, with all the species with reference sequences being detected by the MiSeq sequencing (see below). The new forward (MiFish-E-F) and reverse (MiFish-E-R) primers were designed in an identical region to that of the universal primers, consisting of 21 and 27 bases (table 3) with G/C contents of 52% and 41% and *T*_*m*_ of 54.1°C and 55.2°C, respectively, and were used with MiFish-U in multiplex PCR.

**Table 3.** Nucleotide sequences of the universal primers more specifically designed for the elasmobranchs (sharks and rays; MiFish-E) and base compositions in the selected 160 species (electronic supplementary material, table S2). (Nucleotide differences from MitoFish-U are highlighted with underline in bold. This forward (F) and reverse (R) primer pair amplifies the mid region of the mitochondrial 12S rRNA gene with a mean length of 182 bp (170–185 bp).)

MiFish-E-F	5′-	G	T	**T**	G	G	T	A	A	A	**T**	C	T	C	G	T	G	C	C	A	G	C	-3′						
	A	4	0	0	0	0	0	70	157	157	3	0	0	0	0	0	0	0	0	158	0	0							
	C	0	3	14	0	0	0	32	0	0	6	157	0	157	0	0	0	158	158	0	0	158							
	G	153	0	0	157	157	0	0	0	0	1	0	0	1	158	0	158	0	0	0	158	0							
	T	0	154	143	0	0	157	55	0	0	148	1	158	0	0	158	0	0	0	0	0	0							

MiFish-E-R	3′-	G	T	T	T	G	A	**T**	C	C	T	A	A	T	C	T	A	T	G	G	G	G	T	G	A	T	A	C	-5′
	A	0	160	160	160	0	0	153	0	0	160	0	0	160	0	160	2	160	0	0	2	0	160	0	0	160	0	1	
	C	160	0	0	0	160	0	0	0	0	0	0	0	0	0	0	2	0	160	160	158	8	0	159	0	0	0	0	
	G	0	0	0	0	0	0	7	160	160	0	0	0	0	160	0	4	0	0	0	0	0	0	0	0	0	0	159	
	T	0	0	0	0	0	160	0	0	0	0	160	160	0	0	0	152	0	0	0	0	152	0	1	160	0	160	0	

#### 3.1.3 MiFish-tuna

In addition to newly constructed pairs of the universal primers (MiFish-U/E), preliminary experiments showed that nucleotide differences in the MiFish sequences from tunas (seven species of *Thunnus*) were so small that the bioinformatic pipeline was unable to assign assembled reads to the correct species (see below). We visually inspected the entire mitogenome sequences from the seven species of tunas and found a region with sufficient interspecific variations among constituent species. The newly designed genus-specific forward (MiFish-tuna-F) and reverse (MiFish-tuna-R) primers amplify a portion of the ND5 gene (180 bp), consisting of 22 and 21 bases with G/C contents of 55% and 57% and *T*_*m*_ of 56.9°C and 57.8°C, respectively (see table 3 for primer sequences with adapters).

#### 3.1.4 *In silico* evaluation of interspecific variations

The pairwise edit distances from MiFish and ecoPrimer sequences were calculated for all combinations of 1324 fish species distributed across 59 orders, 319 families and 890 genera (total_1324_C_2_=875 826 pairs) and the resulting distances were sorted into between-order, family, genus and species (table 4).

**Table 4.** Frequency distributions of the interspecific edit distances of the MiFish (above) and ecoPrimer (below) sequences among 1324 fish species deposited in the MitoFish database [16]. (The edit distances are sorted into between-order, family, genus and species. Only edit distances from 0 to less than or equal to 10 are shown.)

MiFish	0	≤1	≤2	≤3	≤4	≤5	≤6	≤7	≤8	≤9	≤10
order	0	0	0	0	0	0	0	0	0	0	0
family	0	3	12	12	12	13	18	28	32	52	68
genus	32	72	98	125	164	201	251	316	377	430	479
species	98	187	239	294	361	413	472	524	591	645	684

ecoPrimer	0	≤1	≤2	≤3	≤4	≤5	≤6	≤7	≤8	≤9	≤10
order	0	0	0	4	12	40	85	147	254	355	465
family	2	14	38	95	163	269	365	466	572	654	736
genus	149	296	412	521	640	732	858	931	1020	1079	1132
species	284	471	603	729	817	885	985	1044	1109	1149	1191

As expected from the size difference between MiFish and ecoPrimer sequences (average lengths 172 bp versus 106 bp), the former appears to have more variation than the latter and also outperforms the latter in unambiguously assigning each taxonomic category (table 4). In particular, MiFish sequences perform well for higher taxonomic categories; for example, all the between-order edit distances are larger than 10 in MiFish sequences, while the smallest one in ecoPrimer sequences is three (four pairs). Also, two pairs of the between-family edit distances from ecoPrimer sequences are zero, indicating that interfamilial discrimination is not feasible for these two pairs. For lower taxonomic categories such as genus and species, MiFish sequences also outperform ecoPrimer sequences in terms of unambiguous taxonomic assignments. For example, the number of pairs with smaller between-genus and species edit distances (e.g. less than or equal to 3) in MiFish sequences are 4.17 and 2.48 times lower than those in ecoPrimer sequences, respectively (table 4).

It appears that MiFish sequences still have inherent limitations to unambiguously assign lower taxonomic categories, such as genus and species. Actually, there are 32 and 98 between-genus and specific pairs with the edit distances of zero, respectively (table 4). For those taxonomic groups with no or a few nucleotide differences in MiFish sequences, we need to develop new molecular markers that contain sufficient information to discriminate constituent species. Development of the new marker for correct species assignments of tunas in this study (MiFish-tuna) represents a good example of such a case (see below).

It should also be noted that those zero distances in the intergeneric comparisons from MiFish sequences (total 32 pairs) are restricted mostly to specific groups of fishes, such as Cichlidae (cichlids; 14 pairs) and Istiophoridae (billfishes; 14 pairs), whose limited genetic divergences in mtDNA are well established (and sometimes misleading owing to gene introgression) compared with their distinct morphological divergences [38–40]. The remaining four pairs include that of Cyprinidae (carp and minnow), Engraulidae (anchovy), Mormyridae (freshwater elephantfish) and Mirapinnidae (hairyfish), all of which are under taxonomic revisions at various taxonomic categories [41–44]. Actually, a recent study [42] demonstrated that members of the latter family Mirapinnidae simply represent larval stages of the different whalefish families, indicating that current fish taxonomy is still in a state of flux.

### 3.2 Primer testing with eDNA from aquarium

#### 3.2.1 Library preparation for metabarcoding

We first tested MiFish-U primers (without adapter sequences) using eDNA from the aquarium tanks in preliminary experiments and observed consistent amplifications across different samples on an agarose gel stained with ethidium bromide (results not shown). The PCR bands from those amplifications, however, were often smearing, with occasional extra bands being observed outside the expected size of the products (*ca* 220 bp).

Following the partial success of PCR using eDNA, we constructed MiFish-U primers for the first PCR by appending adapter sequences at their 5′-ends (figure 2; for primer sequences, see table 5). Optimal experimental conditions for the first PCR with these primers were achieved through trial and error, and we found that choice of a PCR kit (KAPA HiFi HotStart ReadyMix) and associated high-annealing temperatures (65–67°C) in the first PCR are the two most important factors contributing to successful amplifications showing distinct single PCR bands on the agarose gel.

**Table 5.** A list of primers for the first and second PCR used in the paired-end library preparation for the MiSeq analyses; indices (=barcodes) are highlighted with an underline. (Note that those index sequences for the reversal primers (R) are read by MiSeq on the opposite strand and should be reverse/complement in the sample sheet for MiSeq runs.)

primer	sequence (5′–3′)
universal primers for the first PCR	
MiFish-U-F	ACACTCTTTCCCTACACGACGCTCTTCCGATCTNNNNNNGTCGGTAAAACTCGTGCCAGC
MiFish-U-R	GTGACTGGAGTTCAGACGTGTGCTCTTCCGATCTNNNNNNCATAGTGGGGTATCTAATCCCAGTTTG
MiFish-E-F	ACACTCTTTCCCTACACGACGCTCTTCCGATCTNNNNNNGTTGGTAAATCTCGTGCCAGC
MiFish-E-R	GTGACTGGAGTTCAGACGTGTGCTCTTCCGATCTNNNNNNCATAGTGGGGTATCTAATCCTAGTTTG
taxon-specific primers for the first PCR	
MiFish-tuna-ND5-F	ACACTCTTTCCCTACACGACGCTCTTCCGATCTNNNNNNATGTCCTTCCTCCTTATCGGCTG
MiFish-tuna-ND5-R	GTGACTGGAGTTCAGACGTGTGCTCTTCCGATCTNNNNNNTTGCCAGTGGCAGCTACGATC
forward primers for the second PCR (A series)	
2nd_PCR_F_A501	AATGATACGGCGACCACCGAGATCTACACTGAACCTTACACTCTTTCCCTACACGACGCTCTTCCGATCT
2nd_PCR_F_A502	AATGATACGGCGACCACCGAGATCTACACTGCTAAGTACACTCTTTCCCTACACGACGCTCTTCCGATCT
2nd_PCR_F_A503	AATGATACGGCGACCACCGAGATCTACACTGTTCTCTACACTCTTTCCCTACACGACGCTCTTCCGATCT
2nd_PCR_F_A504	AATGATACGGCGACCACCGAGATCTACACTAAGACACACACTCTTTCCCTACACGACGCTCTTCCGATCT
2nd_PCR_F_A505	AATGATACGGCGACCACCGAGATCTACACCTAATCGAACACTCTTTCCCTACACGACGCTCTTCCGATCT
2nd_PCR_F_A506	AATGATACGGCGACCACCGAGATCTACACCTAGAACAACACTCTTTCCCTACACGACGCTCTTCCGA
2nd_PCR_F_A507	AATGATACGGCGACCACCGAGATCTACACTAAGTTCCACACTCTTTCCCTACACGACGCTCTTCCGATCT
2nd_PCR_F_A508	AATGATACGGCGACCACCGAGATCTACACTAGACCTAACACTCTTTCCCTACACGACGCTCTTCCGATCT
forward primers for the second PCR (D series)	
2nd_PCR_F_D501	AATGATACGGCGACCACCGAGATCTACACTATAGCCTACACTCTTTCCCTACACGACGCTCTTCCGATCT
2nd_PCR_F_D502	AATGATACGGCGACCACCGAGATCTACACATAGAGGCACACTCTTTCCCTACACGACGCTCTTCCGATCT
2nd_PCR_F_D503	AATGATACGGCGACCACCGAGATCTACACCCTATCCTACACTCTTTCCCTACACGACGCTCTTCCGATCT
2nd_PCR_F_D504	AATGATACGGCGACCACCGAGATCTACACGGCTCTGAACACTCTTTCCCTACACGACGCTCTTCCGATCT
2nd_PCR_F_D505	AATGATACGGCGACCACCGAGATCTACACAGGCGAAGACACTCTTTCCCTACACGACGCTCTTCCGATCT
2nd_PCR_F_D506	AATGATACGGCGACCACCGAGATCTACACTAATCTTAACACTCTTTCCCTACACGACGCTCTTCCGATCT
2nd_PCR_F_D507	AATGATACGGCGACCACCGAGATCTACACCAGGACGTACACTCTTTCCCTACACGACGCTCTTCCGATCT
2nd_PCR_F_D508	AATGATACGGCGACCACCGAGATCTACACGTACTGACACACTCTTTCCCTACACGACGCTCTTCCGATCT
reverse primers for the second PCR (A series)	
2nd_PCR_R_A701	CAAGCAGAAGACGGCATACGAGATGTCGTGATGTGACTGGAGTTCAGACGTGTGCTCTTCCGATCT
2nd_PCR_R_A702	CAAGCAGAAGACGGCATACGAGATACCACTGTGTGACTGGAGTTCAGACGTGTGCTCTTCCGATCT
2nd_PCR_R_A703	CAAGCAGAAGACGGCATACGAGATTGGATCTGGTGACTGGAGTTCAGACGTGTGCTCTTCCGATCT
2nd_PCR_R_A704	CAAGCAGAAGACGGCATACGAGATCCGTTTGTGTGACTGGAGTTCAGACGTGTGCTCTTCCGATCT
2nd_PCR_R_A705	CAAGCAGAAGACGGCATACGAGATTGCTGGGTGTGACTGGAGTTCAGACGTGTGCTCTTCCGATCT
2nd_PCR_R_A706	CAAGCAGAAGACGGCATACGAGATGAGGGGTTGTGACTGGAGTTCAGACGTGTGCTCTTCCGATCT
2nd_PCR_R_A707	CAAGCAGAAGACGGCATACGAGATAGGTTGGGGTGACTGGAGTTCAGACGTGTGCTCTTCCGATCT
2nd_PCR_R_A708	CAAGCAGAAGACGGCATACGAGATGTGTGGTGGTGACTGGAGTTCAGACGTGTGCTCTTCCGATCT
2nd_PCR_R_A709	CAAGCAGAAGACGGCATACGAGATTGGGTTTCGTGACTGGAGTTCAGACGTGTGCTCTTCCGATCT
2nd_PCR_R_A710	CAAGCAGAAGACGGCATACGAGATTGGTCACAGTGACTGGAGTTCAGACGTGTGCTCTTCCGATCT
2nd_PCR_R_A711	CAAGCAGAAGACGGCATACGAGATTTGACCCTGTGACTGGAGTTCAGACGTGTGCTCTTCCGATCT
2nd_PCR_R_A712	CAAGCAGAAGACGGCATACGAGATCCACTCCTGTGACTGGAGTTCAGACGTGTGCTCTTCCGATCT
reverse primers for the second PCR (D series)	
2nd_PCR_R_D701	CAAGCAGAAGACGGCATACGAGATCGAGTAATGTGACTGGAGTTCAGACGTGTGCTCTTCCGATCT
2nd_PCR_R_D702	CAAGCAGAAGACGGCATACGAGATTCTCCGGAGTGACTGGAGTTCAGACGTGTGCTCTTCCGATCT
2nd_PCR_R_D703	CAAGCAGAAGACGGCATACGAGATAATGAGCGGTGACTGGAGTTCAGACGTGTGCTCTTCCGATCT
2nd_PCR_R_D704	CAAGCAGAAGACGGCATACGAGATGGAATCTCGTGACTGGAGTTCAGACGTGTGCTCTTCCGATCT
2nd_PCR_R_D705	CAAGCAGAAGACGGCATACGAGATTTCTGAATGTGACTGGAGTTCAGACGTGTGCTCTTCCGATCT
2nd_PCR_R_D706	CAAGCAGAAGACGGCATACGAGATACGAATTCGTGACTGGAGTTCAGACGTGTGCTCTTCCGATCT
2nd_PCR_R_D707	CAAGCAGAAGACGGCATACGAGATAGCTTCAGGTGACTGGAGTTCAGACGTGTGCTCTTCCGATCT
2nd_PCR_R_D708	CAAGCAGAAGACGGCATACGAGATGCGCATTAGTGACTGGAGTTCAGACGTGTGCTCTTCCGATCT
2nd_PCR_R_D709	CAAGCAGAAGACGGCATACGAGATCATAGCCGGTGACTGGAGTTCAGACGTGTGCTCTTCCGATCT
2nd_PCR_R_D710	CAAGCAGAAGACGGCATACGAGATTTCGCGGAGTGACTGGAGTTCAGACGTGTGCTCTTCCGATCT
2nd_PCR_R_D711	CAAGCAGAAGACGGCATACGAGATGCGCGAGAGTGACTGGAGTTCAGACGTGTGCTCTTCCGATCT
2nd_PCR_R_D712	CAAGCAGAAGACGGCATACGAGATCTATCGCTGTGACTGGAGTTCAGACGTGTGCTCTTCCGATCT

Based on the above empirical observations, we constructed 14 dual-indexed, paired-end libraries through two-step tailed PCR (figure 2) for two to five water samples from each of the four aquarium tanks.

#### 3.2.2 MiSeq sequencing and data analysis

The MiSeq paired-end sequencing (2× 150 bp) of the 14 libraries, together with another 129 libraries (total number of libraries =143), yielded a total of 14.86 million reads, with an average of 95.0% base calls being Phred quality scores of more than or equal to 30.0 (Q30; error rate =0.1% or base call accuracy =99.9%). This run was highly successful considering that the quality scores specified by Illumina is more than 80% bases higher than Q30 at 2×150 bp (Illumina Publication no. 770-2011-001 as of 27 May 2014).

After demultiplexing and subsequent pre-processing of the raw data from MiSeq, the outputs were subjected to the BLAST searches for taxonomic assignment. In total, 4 322 882 reads were assigned to fish species with more than or equal to 97% identity to reference sequences in the custom database. Of these, 4 053 184 (93.4%) are identified as those fishes contained in one of the four tanks (hereafter called ‘tank species’) and the remaining 286 446 (6.6%) are derived from ‘non-tank species’ (table 6), discussed below.

**Table 6.** A summary of the BLAST searches for the four aquarium tanks.

number of reads^*a*^	total	Kuroshio	tropical fish	deep-sea	mangrove
more than or equal to 97% identity with reference sequences (number of libraries)	4 322 882 (14)	2 568 008 (5)	1 299 788 (4)	259 191 (3)	212 643 (2)
tank fish	4 053 184 (93.4%)	2 375 892 (92.5%)	1 237 546 (95.2%)	245 201 (94.6%)	194 545 (91.5%)
non-tank fish	286 446 (6.6%)	192 116 (7.5%)	62 242 (4.8%)	13 990 (5.4%)	18 098 (8.5%)
number of tank species	249	75	159	15	8
number of tank species with reference sequences	180	63	105	13	8
number of tank species detected in MiSeq analysis	168 (93.3%)	61 (96.8%)	95 (90.5%)	13 (100%)	8 (100%)
water volumes of tank (m^3^)	8465	7500	700	230	35.6

^*a*^Those reads with less than 97% sequence identity are excluded from the above table for simplicity. They are 285 172 reads in total; 57 572 reads from the Kuroshio, 222 897 reads from the tropical fish, 1093 reads from the deep-sea and 3610 reads from the mangrove tanks, respectively.

According to the unpublished monthly report from the aquarium, the four tanks harboured a diverse range of 249 fish species distributed across 64 families and 146 genera at the time of sampling. Of these 249 species, we confirmed that 180 species have reference sequences in the custom database (tables 7 and 8) and detected eDNA from 168 species (93.3%; table 6). In the following, we describe and discuss results from the metabarcoding analyses of each tank separately.

**Table 7.** Taxonomic composition and read numbers of the 168 species detected in MiSeq analyses of eDNA samples from the four aquarium tanks. (Only those species contained in the respective tanks with reference sequences in the custom database are shown.)

higher classification^*a*^	species	total	Kuroshio	tropical	deep	mangrove
Class Chondrichthyes (cartilaginous fishes)						
Subclass Elasmobranchii						
Subdivision Selachii (sharks)						
Order Orectolobiformes						
Family Orectolobidae	*Stegostoma fasciatum*	788	788	0	0	0
Family Hemiscyllidae	*Chiloscyllium punctatum*	21	0	21	0	0
Family Gygliomostomatidae	*Nebrius ferrugineus*	997	997	0	0	0
Family Rhincodontidae	*Rhincodon typus*	6864	6864	0	0	0
Order Carcharhiniformes						
Family Triakidae	*Mustelus manazo*	38	0	0	38	0
Family Carcharhinidae	*Carcharhinus leucas*	16	16	0	0	0
	*Carcharhinus plumbeus*	816	816	0	0	0
	*Galeocerdo cuvier*	2236	2236	0	0	0
	*Negaprion acutidens*	383	383	0	0	0
	*Triaenodon obesus*	24	24	0	0	0
Order Squaliformes						
Family Squalidae	*Cirrhigaleus barbifer*	177	0	0	177	0
	*Squalus brevirostris*^*b*^	129	0	0	129	0
Order Pristiophoriformes						
Family Pristiophoridae	*Pristiophorus japonicus*	9484	0	0	9484	0
Subdivision Batoidea (rays)						
Order Rajiformes						
Family Rhinidae	*Rhina ancylostoma*	614	614	0	0	0
	*Rhynchobatus djiddensis*	10 405	10 405	0	0	0
Order Myliobatifrormes						
Family Dasyatidae	*Dasyatis ushiyei*	265	265	0	0	0
	*Himantura fai*	2799	2799	0	0	0
	*Himantura uarnak*	3584	3584	0	0	0
	*Urogymnus asperrimus*	577	577	0	0	0
Family Myliobatidae	*Aetobatus narinari*	1167	1167	0	0	0
	*Manta alfredi*	7701	7701	0	0	0
	*Rhinoptera javanica*^*c*^	5464	5464	0	0	0
Class Actinopterygii (ray-finned fishes)						
Subclass Neopterygii						
Division Teleostei						
Order Elopiformes						
Family Elopidae	*Elops hawaiensis*	3040	3040	0	0	0
Order Anguilliformes						
Family Muraenidae	*Gymnothorax isingteena*	739	0	739	0	0
Order Beryciformes						
Family Trachichthyidae	*Gephyroberyx japonicus*	3240	0	0	3240	0
Family Holocentridae	*Myripristis berndti*	148	0	148	0	0
	*Neoniphon sammara*	149	0	149	0	0
	*Ostichthys japonicus*	2506	0	0	2506	0
	*Sargocentron rubrum*	766	0	766	0	0
Order Mugiliformes						
Family Mugilidae	*Ellochelon vaigiensis*	491	0	0	0	491
Order Gasterosteiformes						
Suborder Syngnathoidei						
Family Fistulariidae	*Fistularia commersonii*	2458	0	2458	0	0
Family Centriscidae	*Aeoliscus strigatus*	404	0	404	0	0
Order Scorpaeniformes						
Suborder Scorpaenoidei						
Family Scorpaenidae	*Pterois volitans*	795	0	795	0	0
Order Perciformes						
Suborder Percoidei						
Family Serranidae	*Cephalopholis argus*	317	0	317	0	0
	*Cephalopholis sonnerati*	2403	0	2403	0	0
	*Cephalopholis urodeta*	2365	0	2365	0	0
	*Epinephelus bruneus*	983	983	0	0	0
	*Epinephelus coioides*	8639	0	8639	0	0
	*Epinephelus fasciatus*	5626	0	5626	0	0
	*Epinephelus lanceolatus*	67 311	21 026	46 285	0	0
	*Epinephelus maculatus*	5124	0	5124	0	0
	*Epinephelus tukula*	17 116	3579	13 537	0	0
	*Plectropomus leopardus*	3758	0	3758	0	0
	*Variola louti*	286	0	286	0	0
Family Priacanthidae	*Priacanthus hamrur*	16 641	0	16 641	0	0
Family Apogonidae	*Sphaeramia orbicularis*	22 946	0	0	0	22 946
Family Scombropidae	*Scombrops gilberti*^*d*^	649	0	0	649	0
Family Coryphaenidae	*Coryphaena hippurus*	7143	7143	0	0	0
Family Echeneidae	*Echeneis naucrates*	9187	9187	0	0	0
Family Carangidae	*Alectis ciliaris*	420	420	0	0	0
	*Alectis indica*	6071	6071	0	0	0
	*Alepes vari*	19 433	19 433	0	0	0
	*Carangichthys dinema*	532	532	0	0	0
	*Caranx ignobilis*	51 693	51 693	0	0	0
	*Caranx melampygus*	55 111	55 111	0	0	0
	*Caranx papuensis*	6029	6029	0	0	0
	*Caranx sexfasciatus*	48 578	48 578	0	0	0
	*Decapterus muroadsi*	1735	1735	0	0	0
	*Elagatis bipinnulata*	58 279	58 279	0	0	0
	*Gnathanodon speciosus*	22 634	22 634	0	0	0
	*Selar crumenophthalmus*	3985	3985	0	0	0
	*Seriola dumerili*	19 935	19 935	0	0	0
	*Seriola rivoliana*	16 863	16 863	0	0	0
	*Trachinotus blochii*	19 129	19 129	0	0	0
	*Uraspis uraspis*	200	200	0	0	0
Family Emmelichthyidae	*Erythrocles schlegelii*	24 447	0	0	24 447	0
Family Lutjanidae	*Aprion virescens*	2217	2217	0	0	0
	*Etelis carbunculus*	9747	0	0	9747	0
	*Etelis coruscans*^*e*^	19 271	0	0	19 271	0
	*Lutjanus bohar*	13 220	3667	9553	0	0
	*Lutjanus decussatus*	179	0	179	0	0
	*Lutjanus fulvus*	4207	0	4207	0	0
	*Lutjanus kasmira*	75 436	2476	72 960	0	0
	*Lutjanus monostigma*	7134	0	7134	0	0
	*Lutjanus sebae*	2477	0	2477	0	0
Family Caesionidae	*Caesio caerulaurea*	10 175	10 175	0	0	0
	*Caesio cuning*	8557	7886	671	0	0
	*Caesio teres*	57 962	25 958	32 004	0	0
	*Pterocaesio marri*	289 474	245 181	44 293	0	0
	*Pterocaesio tile*	97 437	97 437	0	0	0
Family Lobotidae	*Lobotes surinamensis*	29	0	29	0	0
Family Haemulidae	*Diagramma picta*	16 101	0	16 101	0	0
	*Plectorhinchus lineatus*	35 231	0	35 231	0	0
Family Lethrinidae	*Gnathodentex aureolineatus*	25 714	0	25 714	0	0
	*Gymnocranius euanus*	293	293	0	0	0
	*Lethrinus microdon*	3102	3102	0	0	0
	*Lethrinus nebulosus*	44 356	33 466	10 890	0	0
	*Lethrinus olivaceus*	3135	3135	0	0	0
	*Lethrinus ornatus*	779	779	0	0	0
Family Mullidae	*Parupeneus pleurostigma*	647	0	647	0	0
Family Pempheridae	*Pempheris schwenkii*	7113	0	7113	0	0
Family Monodactylidae	*Monodactylus argenteus*	133 612	0	0	0	133 612
Family Toxotidae	*Toxotes chatareus*	16 822	0	0	0	16 822
Family Kyphsidae	*Girella mezina*	5240	0	5 240	0	0
Family Chaetodontidae	*Chaetodon auriga*	2644	0	2644	0	0
	*Chaetodon auripes*	41 991	0	41 991	0	0
	*Chaetodon lunula*	2959	0	2959	0	0
	*Chaetodon vagabundus*	2495	0	2495	0	0
	*Hemitaurichthys polylepis*	1848	0	1848	0	0
	*Heniochus diphreutes*	706	0	706	0	0
Family Pomacanthidae	*Pomacanthus semicirculatus*	1100	0	1100	0	0
Family Pentacerotidae	*Pentaceros japonicus*	13 087	0	0	13 087	0
Family Kuhliidae	*Kuhlia mugil*	1275	0	1275	0	0
Family Cirrhitidae	*Paracirrhites forsteri*	707	0	707	0	0
Family Cheilodactylidae	*Cheilodactylus zonatus*	1983	0	1983	0	0
Suborder Labroidei						
Family Pomacentridae	*Abudefduf sexfasciatus*	98 622	0	98 622	0	0
	*Abudefduf sordidus*	903	0	903	0	0
	*Abudefduf vaigiensis*	4216	0	4216	0	0
	*Amblyglyphidodon curacao*^*f*^	74 516	0	74 516	0	0
	*Amphiprion frenatus*	674	0	674	0	0
	*Chromis atripectoralis*	387	0	387	0	0
	*Chromis viridis*	853	0	853	0	0
	*Chrysiptera cyanea*	2236	0	2236	0	0
	*Neopomacentrus taeniurus*	1113	0	0	0	1113
	*Pomacentrus amboinensis*^*g*^	293	0	293	0	0
Family Labridae	*Bodianus bilunulatus*	10 489	0	10 489	0	0
	*Cheilinus undulatus*	31 336	0	31 336	0	0
	*Choerodon schoenleinii*	45 558	0	45 558	0	0
	*Coris aygula*	1292	0	1292	0	0
	*Coris gaimard*	1433	0	1433	0	0
	*Halichoeres marginatus*	337	0	337	0	0
	*Hologymnosus doliatus*	170	0	170	0	0
	*Iniistius pavo*	532	0	532	0	0
	*Labrichthys unilineatus*	289	0	289	0	0
	*Labroides dimidiatus*	1333	0	1333	0	0
	*Oxycheilinus unifasciatus*	337	0	337	0	0
	*Thalassoma hardwicke*	1718	0	1718	0	0
	*Thalassoma lutescens*	6028	0	6028	0	0
Family Scaridae	*Bolbometopon muricatum*	66	0	66	0	0
	*Cetoscarus bicolor*	145	0	145	0	0
	*Chlorurus microrhinos*	4297	0	4297	0	0
	*Chlorurus sordidus*	3701	0	3701	0	0
	*Scarus frenatus*	3855	0	3855	0	0
	*Scarus ghobban*	134 283	0	134 283	0	0
	*Scarus rivulatus*	564	0	564	0	0
	*Scarus schlegeli*	39 908	0	39 908	0	0
Suborder Trachinoidei						
Family Pinguipedidae	*Parapercis pacifica*	516	0	516	0	0
Suborder Gobioidei						
Family Gobiidae	*Periophthalmus argentilineatus*	928	0	0	0	928
Suborder Acanthuroidei						
Family Ephippidae	*Platax orbicularis*	60 493	0	60 493	0	0
Family Scatophagidae	*Scatophagus argus*	9422	0	0	0	9422
Family Siganidae	*Siganus doliatus*	5628	0	5628	0	0
	*Siganus guttatus*	9211	0	0	0	9211
	*Siganus unimaculatus*	10 521	0	10 521	0	0
Family Zanclidae	*Zanclus cornutus*	8991	0	8991	0	0
Family Acanthuridae	*Acanthurus blochii*	35 342	0	35 342	0	0
	*Acanthurus dussumieri*	19 158	0	19 158	0	0
	*Acanthurus nigricauda*	500	0	500	0	0
	*Acanthurus nigrofuscus*	16 988	0	16 988	0	0
	*Acanthurus olivaceus*	7957	0	7957	0	0
	*Acanthurus xanthopterus*	23 671	0	23 671	0	0
	*Ctenochaetus striatus*	7742	0	7742	0	0
	*Naso hexacanthus*	66 487	572	65 915	0	0
	*Zebrasoma flavescens*	24 888	0	24 888	0	0
Suborder Scombroidei						
Family Gempylidae	*Thyrsitoides marleyi*	150 624	0	0	150 624	0
Family Scombridae	*Auxis thazard thazard*	929	929	0	0	0
	*Euthynnus affinis*	50 100	50 100	0	0	0
	*Grammatorcynus bilineatus*	5605	5605	0	0	0
	*Gymnosarda unicolor*	27 267	27 267	0	0	0
	*Katsuwonus pelamis*	123 814	123 814	0	0	0
	*Rastrelliger kanagurta*	966 420	966 420	0	0	0
	*Thunnus albacares*^*h*^	241 171	241 171	0	0	0
	*Thunnus orientalis*^*i*^	103 957	103 957	0	0	0
Suborder Stromateoidei						
Family Centrolophidae	*Hyperoglyphe japonica*	11 802	0	0	11 802	0
Order Tetraodontiformes						
Suborder Balistoidei						
Family Balistidae	*Melichthys vidua*	1008	0	1008	0	0
	*Odonus niger*	3607	0	3607	0	0
Family Monacanthidae	*Rhinecanthus verrucosus*^*j*^	886	0	886	0	0
Suborder Tetraodontoidei						
Family Tetraodontidae	*Arothron hispidus*	30 458	0	30 458	0	0
Family Diodontidae	*Diodon hystrix*	294	0	294	0	0

^*a*^Classification follows ‘Fishes of the World’ [32].

^*b*^96.7% identity with a congener *Squalus mitsukurii.*

^*c*^95.0% identity with the reference sequence.

^*d*^100% identity with a congener *Scombrops gilberti.*

^*e*^No reference sequence, but 95.3% identity with a congener *Etelis coruscans.*

^*f*^100% identity with a congener *Amblyglyphidodon aureus.*

^*g*^98.8% identity with a congener *Pomacentrus albicaudatus.*

^*h*^Total read number of those tuna species identified as *T. albacares*, *T. maccoyii*, *T. thynnus* and *T. tonggol* (see table 9).

^*i*^Total read number of those tuna species identified as *T. alalungai* and *T. orientalis* (see table 9).

^*j*^100% identity with a congener *Rhinecanthus aculeatus*.

**Table 8.** A list of species with reference sequences in the custom database, but undetected in the MiSeq analyses.

tank	family	species
Kuroshio	Carangidae	*Carangoides orthogrammus*
		*Pseudocaranx dentex*
tropical fish	Dactylopteridae	*Dactyloptena orientalis*
	Serranidae	*Epinephelus merra*
	Lutjanidae	*Lutjanus stellatus*
	Mullidae	*Parupeneus multifasciatus*
	Chaetodontidae	*Forcipiger flavissimus*
	Pomacentridae	*Amphiprion ocellaris*
	Labridae	*Oxycheilinus digramma*
	Scaridae	*Scarus psittacus*
	Acanthuridae	*Zebrasoma scopas*
	Balistidae	*Balistapus undulatus*

#### 3.2.3 Kuroshio tank

The Kuroshio tank (figure 1*a*) is designed for exhibiting marine megafauna, with dimensions (*L*×*W*×*D*) of 35 m×27 m×10 m, large enough (7500 m^3^) to accommodate a number of mature whale sharks (more than 10 m in total length). It predominantly keeps large-sized fishes characteristic to areas around the Kuroshio, one of the western boundary currents flowing northeastwards along the entire length of Japan, including the Okinawa Islands. Preliminary experiments showed that the exclusive use of an MiFish-U primer pair was unable to detect most species of the elasmobranchs (including whale sharks); subsequent development of MiFish-E primers and application of multiplex PCR (MiFish-U/E), however, enabled us to detect all species of the elasmobranchs contained in the tank (table 7).

Out of the 63 fish species with reference sequences in the custom database, we detected 61 species (96.8%) including 17 and 44 species of elasmobranchs and teleosts, respectively, which are collectively distributed across 17 families and 44 genera (table 7). The two undetected species (3.2%) are carangids (*Carangoides orthogrammus* and *Pseudocaranx dentex*; table 8) and we visually confirmed their presence in the tank. There were no extra carangid sequences referable to those two species in the MiSeq outputs, suggesting that they may represent an example of false negative in our metabarcoding analyses.

**Figure 3.**
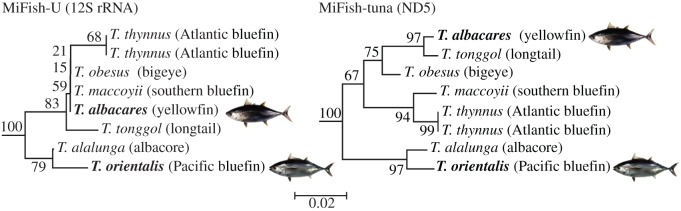
Neighbour-joining trees of the seven species of tunas based on the amplified regions with multiplex PCR using MiFish-U (12S rRNA gene) and MiFish-tuna (ND5 gene) primers. Two species contained in the Kuroshio tank (yellowfin and Pacific bluefin) are highlighted in bold. Distances are calculated by using the Kimura's two-parameter model of base substitution with gaps being completely deleted. Numerals beside the internal branches are bootstrap probabilities based on 300 pseudo-replicates, and branch lengths are proportional to substitutions per site. Photos of the two tuna species are courtesy of H. Senou (Kanagawa Prefectural Museum of Natural History).

Although yellowfin and Pacific bluefin are the only tuna species contained in the Kuroshio tank, our custom bioinformatic pipeline erroneously assigned assembled reads into supposedly six tuna species (table 9). This is apparently owing to small interspecific nucleotide differences among the seven species of tunas, with a mean pairwise *p*-distance of only 2.22 (range 0–5; figure 3) in the MiFish sequences. To resolve this erroneous taxonomic assignment, we developed new genus-specific primers (MiFish-tuna) that amplify a segment of the mitochondrial ND5 gene (180 bp). The amplified region has sufficient interspecific nucleotide variation, with a mean pairwise *p*-distance of 11.1 (range 2–16), and library preparations using multiplex PCR (simultaneous use of MiFish-U/E and MiFish-tuna) lead to correct assignment of the MiSeq outputs into both tuna species present (table 9). Based on this correct taxonomic assignment, we add those erroneous assignments for southern bluefin + Atlantic bluefin + longtail (1808 + 37 + 152 reads) and albacore (103 957 reads) to those of yellowfin (241 171 reads) and Pacific bluefin (306 reads), respectively (table 7).

**Table 9.** Six species of tunas (genus *Thunnus*) and read numbers (pooled from five samples) detected in MiSeq analyses using the 12S primers only (MiFish-U/E) and 12S + ND5 primers (MiFish-U/E/tuna) in multiplex PCR. (*Thunnus albacares* (yellowfin) and *T. orientalis* (Pacific bluefin) in bold, are contained in the Kuroshio tank and the latter analysis with the ND5 sequences only correctly assigned the two species.)

	12S primers only (MiFish-U/E)	12S + ND5 primers (MiFish-U/E/tuna)	
species (common name)	12S	12S	ND5
*T. alalunga*(albacore)	103 957	15 049	0
***T. albacares*(yellowfin)**	**241 171**	**40 578**	**13 259**
*T. maccoyii* (southern bluefin)	1808	392	0
***T. orientalis*(Pacific bluefin)**	**306**	**0**	**17 174**
*T. thynnus* (Atlantic bluefin)	37	0	0
*T. tonggol* (longtail)	152	14	0

It should be noted that MiFish-U/E primers also amplified eDNA from a non-fish marine vertebrate (spotted dolphin, *Stenella attenuata*) also present in the Kuroshio tank (excluded from table 7). We actually found many reads from the dolphin across the five samples totalling 37 056. A comparison between the primer sequences of MiFish-U-F/R and priming sites of the dolphin (EU557096) indicates that there is only one mismatch in the middle of the forward primers (excluding two T/G bonds), suggesting that the primers are also useful for detecting non-fish vertebrates by accommodating their unique nucleotide variations at the priming sites.

#### 3.2.4 Tropical fish tank

The tropical fish tank (figure 1*b*) exhibits typical coastal environments around Okinawa Island (figure 1*e*,*f*), displaying soft corals and 155 species of reef-associated fishes. Of the 155 fish species, we confirmed reference sequences for 105 species in the custom database (tables 7 and 8) and detected eDNA from the 95 species distributed across 32 families and 65 genera (tables 6 and 7). The detection rate (90.5%) is somewhat lower than those of the other tanks (96.8–100%; table 6) and the 10 undetected species are taxonomically diverse, distributed across 10 families within 10 genera (table 8). We visually recognized the presence of these 10 species in the tank and reconfirmed detection of eDNA from the same families or genera of those 10 species. This suggests that strong PCR bias derived from primer–template mismatches seems unlikely and the lack of eDNA from these 10 fish species may represent false negatives. Note that co-occurrences of multiple species from some of the speciose genera, such as *Epinephelus* (five spp.), *Lutjanus* (six spp.) and *Scarus* (four spp.) (table 7), do not confuse the taxonomic assignments, because all undetected species from these genera show significant nucleotide differences from those congeners (*p*-distance =2.9−16.6%). The detection rate might also be affected by uncertainty in the species identification based on morphology for the tank species and/or for voucher specimens of the reference sequences.

The large species diversity in this tank (155 spp.) also highlights the importance for taxonomic coverage of the reference sequences in the custom database [45], which only attain approximately two-thirds of the tank species (105 spp.). For the tropical fish tank, we subjected 1 524 620 reads to BLAST searches and were unable to assign 222 897 reads (14.6%) into any species with more than or equal to 97% sequence identity (not shown in table 6). Such taxonomically unassignable reads are minor in other tanks, with 57 572 reads (2.2%) in the Kuroshio, 1093 reads (0.5%) in the deep-sea and 3610 reads (1.7%) in the mangrove tanks, respectively. In the latter three tanks, some species showing 95 to less than 97% sequence identity are referable to the tank species when they have congeners in the reference sequences and represent single members of those genera in the respective tanks (see footnotes in table 7). By contrast, such cases are quite rare in the tropical fish tank and presence of multiple confamilial or congeneric species with less than 97% sequence identity hinders further taxonomic assignments.

#### 3.2.5 Deep-sea tank

The deep-sea tank (figure 1*c*) keeps 15 species of benthic and benthopelagic fishes from elasmobranchs to higher teleosts commonly found in slope waters off Okinawa. Of these 15 deep-sea fish species, we confirmed reference sequences for 13 species in the custom database (table 7) and detected all of these 13 species with eDNA (100%; tables 6 and 7).

#### 3.2.6 Mangrove tank

The mangrove tank exhibits the brackish-water mangrove swamps in Okinawa (figure 1*e*), keeping eight species of teleosts common to those environments. We confirmed reference sequences for all of these eight teleosts in the custom database (table 7) and detected eDNA from all of them (100%; tables 6 and 7).

#### 3.2.7 Detection of non-tank species

The most serious pitfall of eDNA is the risk of contamination, which remains among the greatest experimental challenges to this field [45,46]. To avoid such risk, we performed decontamination procedures for laboratory spaces and equipment and physically separated pre- and post-PCR work spaces (see Material and methods), which are known to significantly limit the contamination [47]. Despite these efforts, a total of 286 446 reads (6.6%) were considered as those from non-tank species and most of them may represent false positives from various sources. In a similar metabarcoding study using universal primers, Kelly *et al.* [12] reported that approximately 25.5% of the tank sequences were assigned to taxa not living in the mesocosm tank (non-tank species) at the Monterey Bay Aquarium.

**Figure 4.**
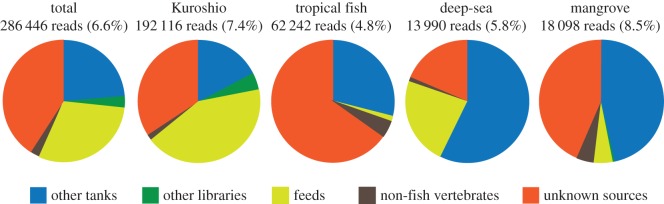
Compositions of the non-tank species (with more than or equal to 97% sequence identity to reference sequences in the custom database) for eDNA from the four tanks in the Okinawa Churaumi Aquarium. Percentages in parentheses are based on the total number of reads with sequence identity of more than or equal to 97% (table 6). For classification of the non-tank species, see text.

Although this study is not designed to rigorously determine the extent of detection rates of such false positives, it would be useful for future eDNA research using the metabarcoding approach to list possible sources of the non-tank species as exogenous DNA with some comments. They can tentatively be classified into: (i) other tank species (62 218 reads; 23.8%); (ii) species from other libraries on the same run (8925 reads; 3.1%); (iii) fish feed (86 204 reads; 30.1%); (iv) non-fish vertebrates (68 735 reads; 2.4%) excluding a spotted dolphin contained in the Kuroshio tank; and (v) unknown (116 264 reads; 42.3%) (figure 4).

One of the most noteworthy examples is detection of non-tank species showing abundant reads in their respective tanks. Those tank species with pooled reads of more than 100 000 were consistently found across other tanks and even from some negative controls, including four species of tunas and mackerels (*Rastrelliger kanagurta*, *Thunnus albacares*, *T. orientalis*, *Katsuwonus pelamis*) plus a fussiler (*Pterocaesio marri*) from the Kuroshio tank, a parrotfish (*Scarus ghobban*) from the tropical fish tank, a snake mackerel (*Thyrsitoides marleyii*) from the deep-sea tank and a moonyfish (*Monodactylus argenteus*) from the mangrove tank. The occasional detection of those reads in the negative controls strongly suggests cross contamination in the laboratory, which seems unavoidable in eDNA studies using PCR amplifications [45]. Although we are unable to pinpoint the experimental step of such contamination, PCR-amplified eDNA during the library preparation, which generate billions of DNA copies in a single reaction, would be the most critical source for large amounts of exogenous DNA [45].

Detection of such non-tank species can be partly explained by re-intake of discharged seawater from the aquarium as it continuously pumps fresh seawater into the facility from the outer reef slope at a depth of 20 m (350 m offshore). Subsequently, the water is directed to various tanks after filtration and is finally led through a drain discharging on the same outer reef slope. Because of the close proximity of the influx and outflow of water (300 m separation), eDNA from non-tank species are likely to occasionally circulate in other tanks as exogenous DNA.

We also encountered putatively exogenous DNA from other libraries (figure 4), which notably consists of subarctic pelagic and benthic fishes from the Bering Sea and adjacent waters (e.g. salmon, northern smoothtongue, sculpins; 8925 reads; 3.1%). All of these dual-indexed paired-end libraries were constructed in other laboratories and cross contamination is highly unlikely. Kircher *et al.* [48] demonstrated such misassignment on the Illumina sequencing platform and the Illumina document (pub. no. 770-2013-046 as of 20 November 2013) recently acknowledged that it can occur during the demultiplexing, a process by which reads are assigned to the sample of origin.

Another source of exogenous DNA includes fish feed (e.g. mackerel, herring, flying fish). They are predominant in the Kuroshio tank (figure 4) where large amounts of those fishes are regularly fed to large-sized elasmobranchs, teleosts and dolphins. We also detected exogenous DNA from non-fish vertebrates (figure 4), mostly from that of humans and domesticated animals such as chickens and pigs, similar to that observed in the mesocosm tank at the Monterey Bay Aquarium [12]. Human eDNA is obviously present from staff diving and maintenance, whereas domesticated animal DNA have frequently been found in chemical reagents [49].

Finally, significant amounts of eDNA from non-tank species are derived from unknown sources other than fish or non-fish vertebrates listed above (116 264 reads; 40.6% among non-tank species and 2.5% among tank + non-tank species). Most of those reads comprise eDNA from non-subtropical marine and freshwater fishes from various localities. It should be noted that such dubious reads are few in eDNA from natural seawater (see below), only comprising 0.58% (5502 reads) of the total reads with more than or equal to 97% sequence identity (954 326 reads). This suggests that seawater from the aquarium tanks contain more exogenous DNA with unknown sources than those from natural environments. Further investigations are needed to more rigorously specify the identity of those dubious sequences from unknown sources.

### 3.3 Primer testing with eDNA from natural seawaters

In addition to the aquarium tanks, we also sampled natural seawater from a rocky coast around the coral reef nearby the aquarium (figure 1*e*,*f*) on two separate days (4 June and 7 November 2014). Using eDNA from four 2 l samples, we prepared four dual-indexed libraries and they were subjected to the MiSeq paired-end sequencing. After demultiplexing and subsequent pre-processing of the raw data from MiSeq, the outputs were subjected to the BLAST searches for taxonomic assignments. In total, 954 326 reads were assigned to fish species with more than or equal to 97% sequence identity to reference sequences in the custom database, of which 948 824 (99.4%) were putatively considered as endogenous eDNA.

From the four water samples, we detected 93 fish species distributed across 36 families and 62 genera (table 10). We confirmed that all of these species occur in the subtropical western North Pacific, although most of them are not particularly obvious and colourful, usually small-sized and/or fossorial reef-associated fishes unsuitable for the aquarium display. Of these 93 fish species, 64 are unique in these samples not detected in the four aquarium tanks and 11 families are new to the taxonomic list (table 10). Unfortunately, there is no background faunal information on fishes in this area, and we are unable to compare the present results with those from previous studies.

**Table 10.** Taxonomic composition and read numbers for the 93 species of teleost fishes detected in the MiSeq analyses of eDNA samples from a rocky coast near the aquarium. (Only those species with identity more than or equal to 97% are shown with numbers of pooled reads from two samples. Asterisks indicate those species also occur in the four aquarium tanks (table 6).)

higher classification^*a*^	species	total	no. 1 (3 June)	no. 2 (7 November)
Order Anguilliformes				
Family Muraenidae	*Echidna nebulosa*	5085	5085	0
	*Echidna polyzona*	111	0	111
	*Gymnothorax pictus*	1141	1141	0
	*Gymnothorax richardsonii*	5850	5850	0
Order Clupeiformes				
Family Clupeidae	*Amblygaster sirm*	94	0	94
Order Gonorynchiformes				
Family Chanidae	*Chanos chanos*	32	0	32
Order Siluriformes				
Family Plotosidae	*Plotosus japonicus*	43	43	0
Order Mugilliformes				
Family Mugilidae	*Chelon affinis*	61	61	0
	*Crenimugil crenilabis*	440	440	0
	*Mugil cephalus*	20 700	20 700	0
Order Atheriniformes				
Family Atherinidae	*Atherinomorus lacunosus*	980	0	980
	*Hypoatherina lunata*	830	0	830
Order Beloniformes				
Family Exocoetidae	*Oxporhamphus convexus*	2489	0	2489
Family Belonidae	*Tylosurus acus melanotus*	6592	0	6592
	*Tylosurus crocodilus*	261 390	261 390	0
Order Beryciformes				
Family Holocentridae	Neoniphon sammara*	4139	4139	0
	Sargocentron punctatissimum*	1579	0	1579
Order Gasterosteiformes				
Suborder Syngnathoidei				
Family Fistulariidae	Fistularia commersonii*	3258	2234	1024
Order Perciformes				
Suborder Percoidei				
Family Serranidae	*Epinephelus polyphekadion*	1408	1408	0
Family Carangidae	Caranx papuensis*	1152	1152	0
	Trachinotus blochii*	1882	1882	0
Family Lutjanidae	*Lutjanus fulviflamma*	11 748	11 748	0
Family Caesionidae	*Pterocaesio chrysozona*	673	0	673
Family Gerreidae	*Gerres equulus*	14	14	0
Family Lethrinidae	Lethrinus nebulosus*	60 040	59 414	626
Family Sparidae	*Acanthopagrus sivicolus*	19 625	16 511	3114
Family Mullidae	*Parupeneus ciliatus*	2865	2865	0
Family Pempheridae	Pempheris schwenkii*	8319	8319	0
Family Kyphosidae	*Kyphosus bigibbus*	1076	28	1048
	*Kyphosus cinerascens*	7861	7861	0
	Girella mezina*	16 978	16 978	0
Family Chaetodontidae	Chaetodon auriga*	27 016	27 016	0
	Chaetodon auripes*	2534	0	2534
	Chaetodon lunula*	6530	6530	0
	*Chaetodon rafflesii*	5780	5780	0
	Chaetodon vagabundus*	1151	1151	0
Suborder Labroidei				
Family Pomacentridae	*Abudefduf septemfasciatus*	139	139	0
	Abudefduf sordidus*	3138	2089	1049
	Abudefduf vaigiensis*	1251	0	1251
	*Cheiloprion labiatus*	27 314	27 314	0
	*Chrysiptera biocellata*	1389	1389	0
	Chrysiptera cyanea*	53 598	52 632	966
	*Chrysiptera glauca*	1085	1085	0
	*Chrysiptera rex*	2493	0	2493
	*Chrysiptera unimaculata*	23 428	23 428	0
	*Plectroglyphidodon lacrymatus*	1 669	0	1669
	*Pomacentrus albicaudatus*	2025	2025	0
	*Stegastes albifasciatus*	27 359	27 359	0
	*Stegastes fasciolatus*	838	0	838
	*Stegastes nigricans*	37 494	37 494	0
Family Labridae	Halichoeres marginatus*	1973	1973	0
	*Halichoeres trimaculatus*	15 601	15 601	0
	*Hemigymnus fasciatus*	26	0	26
	Labroides dimidiatus*	745	745	0
	*Stethojulis bandanensis*	222	222	0
	*Thalassoma bifasciatum*	4453	4453	0
	Thalassoma hardwicke*	1091	1091	0
	Thalassoma lutescens*	2200	294	1906
	*Thalassoma quinquevittatum*	536	0	
Family Scaridae	Chlorurus sordidus*	1777	1329	448
	*Leptoscarus vaigiensis*	280	280	0
	*Scarus forsteni*	1825	1825	0
	*Scarus psittacus*	1189	0	1189
	Scarus rivulatus*	1572	1572	0
	Scarus schlegeli*	2165	0	2165
Suborder Trachinoidei				
Family Pinguipedidae	*Parapercis cylindrica*	751	751	0
Suborder Blennioidei				
Family Blenniidae	*Cirripectes castaneus*	1442	0	1442
	*Cirripectes imitator*	3098	0	3098
	*Istiblennius edentulus*	120 080	118 090	1990
	*Rhadoblennius ellipes*	5585	0	5585
	*Salarias fasciatus*	3919	3248	671
Suborder Gobioidei				
Family Gobiidae	*Bathygobius cocosensis*	1149	0	1149
	*Bathygobius fuscus*	70	70	0
	*Trimma annosum*	148	148	0
	*Trimma caesiura*	279	279	0
Suborder Acanthuroidei				
Family Siganidae	*Siganus fuscescens*	42 912	35 205	7707
Family Acanthuridae	Acanthurus dussumieri*	2453	2453	0
	*Acanthurus leucosternon*	12 954	6492	6462
	*Acanthurus lineatus*	515	0	515
	Acanthurus nigrofuscus*	1516	1516	0
	*Ctenochaetus binotatus*	543	0	543
	Ctenochaetus striatus*	72	0	72
	*Naso lopezi*	0	3611	0
Suborder Scombroidei				
Family Scombridae	Euthynnus affinis*	5147	0	5147
	Rastrelliger kanagurta*	20 734	12 870	7864
	Thunnus albacares*	1190	1190	0
Order Pleuronectiformes				
Suborder Pleuronectoidei				
Family Bothidae	*Bothus pantherinus*	244	244	0
Order Tetraodontiformes				
Suborder Balistoidei				
Family Balistidae	*Balistapus undulatus*	1124	0	1124
Family Monacanthidae	*Cantherhines dumerilii*	875	0	875
	Melichthys vidua*	583	0	583
	*Rhinecanthus aculeatus*	6785	5138	1647
Suborder Tetraodontoidei				
Family Tetraodontidae	*Arothron nigropunctatus*	552	552	0
Family Diodontidae	*Diodon holocanthus*	152	152	0

^*a*^Classification follows ‘Fishes of the World’ [32].

## 4. Concluding remarks

With the use of newly developed universal primers (MiFish-U/E) and a high-throughput NGS platform (Illumina MiSeq) in a metabarcoding approach to fish eDNA, we confirmed the detection of 232 fish species distributed across 70 families and 152 genera from four aquarium tanks and coral reefs in the subtropical western North Pacific. Those 232 species are not only taxonomically diverse, ranging from sharks and rays to higher teleosts, but are also greatly varied in their ecology, including both pelagic and benthic species living in shallow coastal to deep waters. The eDNA metabarcoding approach presented here is non-invasive, more efficient, more cost-effective and more sensitive than the traditional survey methods. It could serve as an alternative (or complementary) tool for biodiversity monitoring that will greatly aid natural resource management and ecological studies of fish communities on larger spatial and temporal scales. In addition to eDNA, this metabarcoding approach is applicable to bulk samples (total DNA), such as those from net collections containing multiple life stages and damaged specimens with no diagnostic characters for species identification. Furthermore, the detection of various mammals suggests the broad applicability of this approach to non-fish vertebrates with slight modifications of primer sequences to accommodate unique nucleotide variations among those organisms.

Nevertheless, there are several methodological challenges that must be addressed before this metabarcoding approach is likely to become a mainstream technology in fish biodiversity research. The first one would be to explore a method that generates a greater diversity of MiFish sequences at a lower cost to avoid PCR dropouts (=false negatives). Those taxa that are prone to the dropouts can potentially skew the relative abundance in eDNA sequences, making it difficult to assess biologically relevant differences across taxonomic groups [34]. Considering stochasticity of individual PCR reactions and PCR bias derived from primer–template mismatches, optimal number of PCR replicates and use of multiple annealing temperatures should be explored to comprehensively detect fish eDNA without the dropouts. In a fungal metabarcoding study, pooling multiple repeated PCRs and using multiple annealing temperatures were recommended to facilitate the recovery of more correct species richness [50].

The second one is false positives that are consistently observed in our metabarcoding analyses of the four aquarium tanks (figure 4). Although sources of the majority of those reads (57.7%) can be identified (e.g. exogenous DNA from other tank species, other libraries, fish feed, non-fish vertebrates), there are a significant number of reads from unknown sources other than the former (42.3%; 2.5% of the total number of reads with more than or equal to 97% sequence identity). Such dubious reads are relatively few in eDNA from the coral reefs near the aquarium (0.58%) and subsequent analyses of eDNA from oceanic waters that are remote from human activities support this observation (results not shown). This also illustrates the limits of the eDNA metabarcoding approach that cannot discriminate sources of eDNA from either exogenous or endogenous origins.

**Figure 5.**
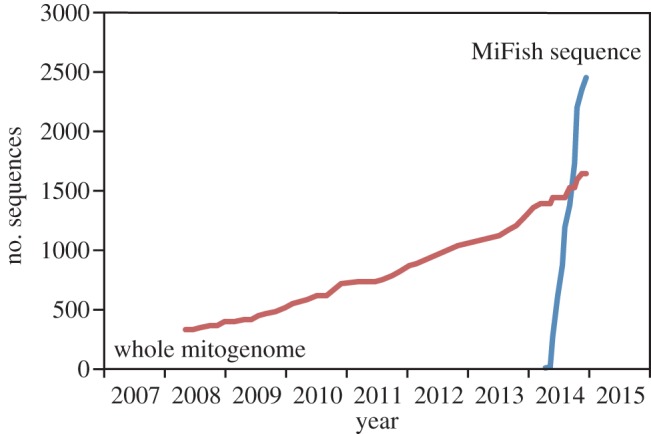
Temporal accumulation of the number of whole mitogenome sequences (*ca* 16 500 bp) curated in MitoFish and the MiFish sequences (*ca* 170 bp) in the custom database. The former data were taken from a change log recorded in MitoFish (http://mitofish.aori.u-tokyo.ac.jp/about/log.html).

The third one is completeness of the reference sequence database, which is indispensable for correct taxonomic assignments. Reference sequences in the custom database used in the present analyses were derived from two data sources. The first one is MitoFish, from which all whole mitogenome sequences (1324 sequences) and partial mitogenome sequences containing MiFish sequences (2953 sequences) were obtained. The second one is supplementary MiFish sequences assembled in M.M.'s laboratory (648 sequences; electronic supplementary material, table S3). In total, it covers approximately 4230 fish species distributed across 457 families and 1827 genera as of 4 October 2014. Obviously, this taxonomic coverage is far from satisfactory, considering the enormous diversity of fishes with at least 27 977 species placed in 515 families and 1827 genera [32]. Nevertheless, total number of fish whole mitogenome sequences in MitoFish [17] has steadily increased since its 2006 onset and the number of original MiFish sequences has increased considerably as a result of recent massive sequencing of the two large tissue collections (figure 5), currently reaching 2364 sequences from a wide variety of fish taxa. Obviously, our custom-made database for newly designed eDNA markers is not compatible to that of other online resources. For example, the Fish Barcode of Life project (http://www.fishbol.org/index.php) currently deposits 107 033 barcoded sequences, which include approximately 10 800 species. Although the increase in mitogenomic sequences will continuously improve this situation, we agree with Thomsen & Willerslev [45] who suggested that, given the massive increase in DNA sequencing cost-efficiency, future DNA reference databases should focus on whole mitochondrial or even nuclear genomes for much wider applications than traditional DNA barcoding.

## Supplementary Material

The following three tables are combined into a single file (pdf) Table S1. A list of fish species used for designing universal primers (MiFish-U) including 880 species placed in 51 orders, 242 families and 623 genera. Table S2. A list of fish species used for designing universal primers specifically designed for elasmobranchs (MiFish-E) including 160 species placed in 12 orders, 39 families and 77 genera. Table S3. A list of fish species used for constructing the custom database for taxonomic assignment using BLAST including 648 sequences from 594 species placed in 36 orders, 162 families and 390 genera.

## Data accessibility

Custom Ruby scripts used in *in silico* evaluation of interspecific variation are available from http://dx.doi.org/10.5061/dryad.54v2q. Raw reads from the MiSeq sequencing are available from the DDBJ Sequence Read Archive (DRR030411–030428). The bioinformatic pipeline from data pre-processing through taxonomic assignment (including Perl scripts) is available from http://dx.doi.org/10.5061/dryad.n245j.

## References

[1] Kelly RP, Port JA, Yamahara KM, Martone RG, Lowell N, Thomsen PF, Mach ME, Bennett M, Prahler E, Caldwell MR 2014 Harnessing DNA to improve environmental management. *Science* 344, 1455–1456. (doi:10.1126/science.1251156) 24970068 10.1126/science.1251156

[2] Ficetola GF, Miaud C, Pompanon F, Taberlet P 2008 Species detection using environmental DNA from water samples. *Biol. Lett.* 4, 423–425. (doi:10.1098/rsbl.2008.0118) 18400683 10.1098/rsbl.2008.0118 PMC2610135

[3] Takahara T, Minamoto T, Doi H 2013 Using environmental DNA to estimate the distribution of an invasive fish species in ponds. *PLoS ONE* 8, e56584 (doi:10.1371/journal.pone.0056584) 23437177 10.1371/journal.pone.0056584 PMC3577852

[4] Takahara T, Minamoto T, Yamanaka H, Doi H, Kawabata Z 2012 Estimation of fish biomass using environmental DNA. *PLoS ONE* 7, e35868 (doi:10.1371/journal.pone.0035868) 22563411 10.1371/journal.pone.0035868 PMC3338542

[5] Sigsgaard EE, Carl H, Møller PR, Thomsen PF 2015 Monitoring the near-extinct European weather loach in Denmark based on environmental DNA from water samples. *Biol. Conserv.* 183, 48–52. (doi:10.1016/j.biocon.2014.11.023)

[6] Wilcox TM, McKelvey KS, Young MK, Jane SF, Lowe WH, Whiteley AR, Schwartz MK 2013 Robust detection of rare species using environmental DNA: the importance of primer specificity. *PLoS ONE* 8, e59520 (doi:10.1371/journal.pone.0059520) 23555689 10.1371/journal.pone.0059520 PMC3608683

[7] Jerde CL, Chadderton WL, Mahon AR, Renshaw MA, Corush J, Budny ML, Mysorekar S, Lodge DM 2013 Detection of Asian carp DNA as part of a Great Lakes basin-wide surveillance program. *Can. J. Fish. Aquat. Sci.* 70, 522–526. (doi:10.1139/cjfas-2012-0478)

[8] Jerde CL, Mahon AR, Chadderton WL, Lodge DM 2011 ‘Sight-unseen’ detection of rare aquatic species using environmental DNA. *Conserv. Lett.* 4, 150–157. (doi:10.1111/j.1755-263X.2010.00158.x)

[9] Mahon AR, Jerde CL, Galaska M, Bergner JL, Chadderton WL, Lodge DM, Hunter ME, Nico LG 2013 Validation of eDNA surveillance sensitivity for detection of Asian carps in controlled and field experiments. *PLoS ONE* 8, e58316 (doi:10.1371/journal.pone.0058316) 23472178 10.1371/journal.pone.0058316 PMC3589332

[10] Minamoto T, Yamanaka H, Takahara T, Honjo MN, Kawabata Z 2012 Surveillance of fish species composition using environmental DNA. *Limnology* 13, 193–197. (doi:10.1007/s10201-011-0362-4)

[11] Thomsen PF, Kielgast J, Iversen LL, Møller PR, Rasmussen M, Willerslev E 2012 Detection of a diverse marine fish fauna using environmental DNA from seawater samples. *PLoS ONE* 7, e41732 (doi:10.1371/journal.pone.0041732) 22952584 10.1371/journal.pone.0041732 PMC3430657

[12] Kelly RP, Port JA, Yamahara KM, Crowder LB 2014 Using environmental DNA to census marine fishes in a large mesocosm. *PLoS ONE* 9, e86175 (doi:10.1371/journal.pone.0086175) 24454960 10.1371/journal.pone.0086175 PMC3893283

[13] Thomsen PF, Kielgast J, Iversen LL, Wiuf C, Rasmussen M, Gilbert MTP, Orlando L, Willerslev E 2012 Monitoring endangered freshwater biodiversity using environmental DNA. *Mol. Ecol.* 21, 2565–2573. (doi:10.1111/j.1365-294X.2011.05418.x) 22151771 10.1111/j.1365-294X.2011.05418.x

[14] Riaz T, Shehzad W, Viari A, Pompanon F, Taberlet P, Coissac E 2011 ecoPrimers: inference of new DNA barcode markers from whole genome sequence analysis. *Nucleic Acids Res.* 39, e145 (doi:10.1093/nar/gkr732) 21930509 10.1093/nar/gkr732 PMC3241669

[15] Taberlet P, Coissac E, Pompanon F, Brochmann C, Willerslev E 2012 Towards next-generation biodiversity assessment using DNA metabarcoding. *Mol. Ecol.* 21, 2045–2050. (doi:10.1111/j.1365-294X.2012.05470.x) 22486824 10.1111/j.1365-294X.2012.05470.x

[16] Rees HC, Maddison BC, Middleditch DJ, Patmore JR, Gough KC 2014 Review: the detection of aquatic animal species using environmental DNA — a review of eDNA as a survey tool in ecology. *J. Appl. Ecol.* 51, 1450–1459. (doi:10.1111/1365-2664.12306)

[17] Iwasaki W 2013 MitoFish and MitoAnnotator: a mitochondrial genome database of fish with an accurate and automatic annotation pipeline. *Mol. Biol. Evol.* 30, 2531–2540. (doi:10.1093/molbev/mst141) 23955518 10.1093/molbev/mst141 PMC3808866

[18] Inoue JG, Miya M, Tsukamoto K, Nishida M 2003 Evolution of the deep-sea gulper eel mitochondrial genomes: large-scale gene rearrangements originated within the eels. *Mol. Biol. Evol.* 20, 1917–1924. (doi:10.1093/molbev/msg206) 12949142 10.1093/molbev/msg206

[19] Katoh K, Toh H 2008 Recent developments in the MAFFT multiple sequence alignment program. *Briefings Bioinform.* 9, 286–298. (doi:10.1093/bib/bbn013) 10.1093/bib/bbn013 18372315

[20] Maddison WP, Maddison DR 2010 Mesquite: a modular system for evolutionary analysis, v. 2.75. See http://mesquiteproject.org.

[21] Palumbi S 1996 Nucleic acids II: the polymerase chain reaction. In *Molecular systematics* (eds D Hillis, C Moritz, B Mable), pp. 205–247. Sunderland, MA: Sinauer Associates.

[22] Kibbe WA 2007 OligoCalc: an online oligonucleotide properties calculator. *Nucleic Acids Res.* 35, W43–W46. (doi:10.1093/nar/gkm234) 17452344 10.1093/nar/gkm234 PMC1933198

[23] Jones NC, Pevzner P 2004 *An introduction to bioinformatics algorithms*. Cambridge, MA: MIT Press.

[24] Sato Y, Kojima K, Nariai N, Yamaguchi-Kabata Y, Kawai Y, Takahashi M, Mimori T, Nagasaki M 2014 SUGAR: graphical user interface-based data refiner for high-throughput DNA sequencing. *BMC Genomics* 15, 664 (doi:10.1186/1471-2164-15-664) 25103311 10.1186/1471-2164-15-664 PMC4133631

[25] Cox MP, Peterson DA, Biggs PJ 2010 SolexaQA: at-a-glance quality assessment of Illumina second-generation sequencing data. *BMC Bioinform.* 11, 485 (doi:10.1186/1471-2105-11-485) 10.1186/1471-2105-11-485 PMC2956736 20875133

[26] Ewing B, Hillier L, Wendl MC, Green P 1998 Base-calling of automated sequencer traces using Phred. I. Accuracy assessment. *Genome Res.* 8, 175–185. (doi:10.1101/gr.8.3.175) 9521921 10.1101/gr.8.3.175

[27] Magoč T, Salzberg SL 2011 FLASH: fast length adjustment of short reads to improve genome assemblies. *Bioinformatics* 27, 2957–2963. (doi:10.1093/bioinformatics/btr507) 21903629 10.1093/bioinformatics/btr507 PMC3198573

[28] Schmieder R, Lim YW, Rohwer F, Edwards R 2010 TagCleaner: Identification and removal of tag sequences from genomic and metagenomic datasets. *BMC Bioinform.* 11, 341 (doi:10.1186/1471-2105-11-341) 10.1186/1471-2105-11-341 PMC2910026 20573248

[29] Cock PJ, Fields CJ, Goto N, Heuer ML, Rice PM 2010 The Sanger FASTQ file format for sequences with quality scores, and the Solexa/Illumina FASTQ variants. *Nucleic Acids Res.* 38, 1767–1771. (doi:10.1093/nar/gkp1137) 20015970 10.1093/nar/gkp1137 PMC2847217

[30] Edgar RC 2010 Search and clustering orders of magnitude faster than BLAST. *Bioinformatics* 26, 2460–2461. (doi:10.1093/bioinformatics/btq461) 20709691 10.1093/bioinformatics/btq461

[31] Camacho C, Coulouris G, Ma N, Papadopoulos J, Bealer K, Madden TL 2009 BLAST+: architecture and applications. *BMC Bioinform.* 10, 421 (doi:10.1186/1471-2105-10-421) 10.1186/1471-2105-10-421 PMC2803857 20003500

[32] Nelson JS 2006 *Fishes of the world*, 4th edn. Hoboken, NJ: John Wiley and Sons.

[33] Ward RD, Hanner R, Hebert PD 2009 The campaign to DNA barcode all fishes, FISH-BOL. *J. Fish Biol.* 74, 329–356. (doi:10.1111/j.1095-8649.2008.02080.x) 20735564 10.1111/j.1095-8649.2008.02080.x

[34] Deagle BE, Jarman SN, Coissac E, Pompanon F, Taberlet P 2014 DNA metabarcoding and the cytochrome c oxidase subunit I marker: not a perfect match. *Biol. Lett.* 10, 20140562 (doi:10.1098/rsbl.2014.0562) 25209199 10.1098/rsbl.2014.0562 PMC4190964

[35] Van de Peer Y, Van den Broeck I, De Rijk P, De Wachter R 1994 Database on the structure of small ribosomal subunit RNA. *Nucleic Acids Res.* 22, 3488–3494. (doi:10.1093/nar/22.17.3488) 7524022 10.1093/nar/22.17.3488 PMC308309

[36] Miya M, Nishida M 1998 Molecular phylogeny and evolution of the deep-sea fish genus *Sternoptyx*. *Mol. Phylogen. Evol.* 10, 11–22. (doi:10.1006/mpev.1997.0479) 10.1006/mpev.1997.0479 9751914

[37] Wang H-Y, Lee S-C 2002 Secondary structure of mitochondrial 12S rRNA among fish and its phylogenetic applications. *Mol. Biol. Evol.* 19, 138–148. (doi:10.1093/oxfordjournals.molbev.a004066) 11801742 10.1093/oxfordjournals.molbev.a004066

[38] Kornfield I, Smith PF 2000 African cichlid fishes: model systems for evolutionary biology. *Annu. Rev. Ecol. Syst.* 2000, 163–196. (doi:10.1146/annurev.ecolsys.31.1.163)

[39] Graves JE, McDowell JR 2003 Stock structure of the world's istiophorid billfishes: a genetic perspective. *Mar. Freshw. Res.* 54, 287–298. (doi:10.1071/MF01290)

[40] Miya M *et al* 2013 Evolutionary origin of the Scombridae (tunas and mackerels): members of a Paleogene adaptive radiation with 14 other pelagic fish families. *PLoS ONE* 8, e73535 (doi:10.1371/journal.pone.0073535) 24023883 10.1371/journal.pone.0073535 PMC3762723

[41] Lavoué S, Sullivan JP 2014 *Petrocephalus boboto* and *Petrocephalus arnegardi*, two new species of African electric fish (Osteoglossomorpha, Mormyridae) from the Congo River basin. *ZooKeys* 400, 43 (doi:10.3897/zookeys.400.6743) 24843255 10.3897/zookeys.400.6743 PMC4023242

[42] Johnson GD, Paxton JR, Sutton TT, Satoh TP, Sado T, Nishida M, Miya M 2009 Deep-sea mystery solved: astonishing larval transformations and extreme sexual dimorphism unite three fish families. *Biol. Lett.* 5, 235–239. (doi:10.1098/rsbl.2008.0722) 19158027 10.1098/rsbl.2008.0722 PMC2667197

[43] Tang KL *et al* 2013 Limits and phylogenetic relationships of East Asian fishes in the subfamily Oxygastrinae (Teleostei: Cypriniformes: Cyprinidae). *Zootaxa* 3681, 101–135. (doi:10.11646/zootaxa.3681.2.1) 25232592 10.11646/zootaxa.3681.2.1

[44] Lavoué S, Konstantinidis P, Chen W-J 2014 Progress in clupeiform systematics. In *Biology* and ecology of sardines and anchovies (ed. K Ganias), pp. 3–42. Broken Sound Parkway NW: CRC Press.

[45] Thomsen PF, Willerslev E 2014 Environmental DNA: an emerging tool in conservation for monitoring past and present biodiversity. *Biol. Conserv.* 183, 4–18. (doi:10.1016/j.biocon.2014.11.019)

[46] Pedersen MW *et al* 2015 Ancient and modern environmental DNA. *Phil. Trans. R. Soc. B.* 370, 20130383 (doi:10.1098/rstb.2013.0383) 25487334 10.1098/rstb.2013.0383 PMC4275890

[47] Willerslev E, Cooper A 2005 Review paper. Ancient DNA. *Proc. R. Soc. B* 272, 3–16. (doi:10.1098/rspb.2004.2813) 10.1098/rspb.2004.2813 PMC1634942 15875564

[48] Kircher M, Sawyer S, Meyer M 2011 Double indexing overcomes inaccuracies in multiplex sequencing on the Illumina platform. *Nucleic Acids Res.* 40, e3 (doi:10.1093/nar/gkr771) 22021376 10.1093/nar/gkr771 PMC3245947

[49] Champlot S, Berthelot C, Pruvost M, Bennett EA, Grange T, Geigl E-M 2010 An efficient multistrategy DNA decontamination procedure of PCR reagents for hypersensitive PCR applications. *PLoS ONE* 5, e13042 (doi:10.1371/journal.pone.0013042) 20927390 10.1371/journal.pone.0013042 PMC2946917

[50] Schmidt P-A, Bálint M, Greshake B, Bandow C, Römbke J, Schmitt I 2013 Illumina metabarcoding of a soil fungal community. *Soil Biol. Biochem.* 65, 128–132. (doi:10.1016/j.soilbio.2013.05.014)

