# Supplementary material for: MiFish, a set of universal PCR primers for metabarcoding environmental DNA from fishes: detection of more than 230 subtropical marine species
Source: R Soc Open Sci. 2015 Jul 22;2(7):150088. doi: 10.1098/rsos.150088 (PMC4632578; doi:10.1098/rsos.150088)
Supplement: The following three tables are combined into a single file (pdf) Table S1. A list of fish species used for designing universal primers (MiFish-U) including 880 species placed in 51 orders, 242 families and 623 genera. Table S2. A list of fish species used for designing universal primers specifically [file rsos150088supp1.pdf]

Contents of the electronic supplementary material

Table S1. A list of fish species used for designing universal primers (MiFish-U) including 880 species placed in 51 orders, 242 families and 623 genera. ········· 1

Table S2. A list of fish species used for designing universal primers specifically designed for elasmobranchs (MiFish-E) including 160 species placed in 12 orders, 39 families and 77 genera. ········· 18

Table S3. A list of fish species used for constructing the custom database for taxonomic assignment using BLAST including 648 sequences from 594 species placed in 36 orders, 162 families and 390 genera. ········· 21

Table S1.
A list of fish species used for designing universal primers (MiFish-U) including 880 species placed in 51 orders, 242 families and 623 genera.

Order	Family	Species	Accession No.
Chimaeriformes	Callorhinchidae	*Callorhinchus callorynchus*	HM147135
		Callorhinchus capensis	HM147136
		Callorhinchus milii	HM147137
	Rhinochimaeridae	*Harriotta raleighana*	HM147140
	Chimaeridae	*Chimaera fulva*	HM147138
		Chimaera monstrosa	AJ310140
Heterodontiformes	Heterodontidae	*Heterodontus francisci*	AJ310141
Orectolobiformes	Hemiscylliidae	*Chiloscyllium griseum*	JQ434458
		Chiloscyllium plagiosum	FJ853422
		Chiloscyllium punctatum	JQ082337
Lamniformes	Mitsukurinidae	*Mitsukurina owstoni*	EU528659
Carchariniformes	Scyliorhinidae	*Scyliorhinus canicula*	Y16067
	Triakidae	*Mustelus manazo*	AB015962
	Carcharhinidae	*Scoliodon laticaudus*	JQ693102
Squaliformes	Squalidae	*Squalus acanthias*	Y18134
Rajiformes	Rajidae	*Amblyraja radiata*	AF106038
		Leucoraja erinacea	JQ034406
		Okamejei kenojei	AY525783
Myliobatiformes	Hexatrygonidae	*Plesiobatis daviesi*	AY597334
Polypteriformes	Polypteridae	*Polypterus ornatipinnis*	U62532
		Polypterus senegalus senegalus	AP004352
Acipenseriformes	Acipenseridae	*Acipenser baerii*	JQ045341
		Acipenser dabryanus	AY510085
		Acipenser gueldenstaedtii	FJ392605
		Acipenser sinensis	EU719645
		Acipenser stellatus	AJ585050
		Acipenser transmontanus	AB042837
		Scaphirhynchus cf. *albus*	AP004354
	Polyodontidae	*Polyodon spathula*	AP004353
		Psephurus gladius	AY571339
Lepisosteiformes	Lepisosteidae	*Atractosteus spatula*	AP004355
		Lepisosteus oculatus	AB042861
		Lepisosteus osseus	DQ536423
Amiiformes	Amiidae	*Amia calva*	AB042952
Hiodontiformes	Hiodontidae	*Hiodon alosoides*	AP004356
		Hiodon tergisus	AP009499
Osteoglossiformes	Osteoglossidae	*Arapaima gigas*	EF523611
		Heterotis niloticus	AP009498
		Osteoglossum bicirrhosum	AB043025
		Pantodon buchholzi	AB043068
		Scleropages formosus	DQ023143
	Notopteridae	*Chitala blanci*	AP008921
		Chitala lopis	AP008922
		Chitala ornata	AP008923
		Notopterus notopterus	AP008924
		Papyrocranus congoensis	AP008926
		Xenomystus nigri	AP008927
	Mormyridae	*Brienomyrus niger*	AP008929
		Genyomyrus donnyi	AP009500
		Gnathonemus petersii	AP008928
		Gymnarchus niloticus	AP008930

		Marcusenius senegalensis	AP011575
		Myomyrus macrops	AP009501
		Paramormyrops gabonensis	AP009614
		Petrocephalus soudanensis	AP009502
Elopiformes	Elopidae	*Elops hawaiensis*	AB051070
		Elops saurus	AP004807
	Megalopidae	*Megalops atlanticus*	AP004808
		Megalops cyprinoides	AB051110
Albuliformes	Albulidae	*Albula glossodonta*	AP002973
		Pterothrissus gissu	AB051197
	Halosauridae	*Aldrovandia affinis*	AP002974
	Notacanthidae	*Notacanthus chemnitzi*	AP002975
Anguilliformes	Anguilllidae	*Anguilla anguilla*	AP007233
		Anguilla australis australis	AP007234
		Anguilla australis schmidti	AP007235
		Anguilla bengalensis labiata	AP007245
		Anguilla bicolor bicolor	AP007236
		Anguilla bicolor pacifica	AP007237
		Anguilla dieffenbachii	AP007240
		Anguilla interioris	AP007241
		Anguilla japonica	AB038556
		Anguilla luzonensis	AB469437
		Anguilla malgumora	AP007238
		Anguilla marmorata	AP007242
		Anguilla megastoma	AP007243
		Anguilla mossambica	AP007244
		Anguilla nebulosa nebulosa	AP007246
		Anguilla obscura	AP007247
		Anguilla reinhardtii	AP007248
		Anguilla rostrata	AP007249
		Anguilla sp. HYT-2008	AB469437
	Heterenchelyidae	*Pythonichthys microphthalmus*	AP010842
	Moringuidae	*Moringua edwardsi*	AP010840
		Moringua microchir	AP010841
	Chlopsidae	*Kaupichthys hyoproroides*	AP010845
		Robinsia catherinae	AP010846
		Thalassenchelys sp. Tht2	AP010867
	Myrocongridae	*Myroconger compressus*	AP010847
	Muraenidae	*Anarchias* sp. Ansp	AP010843
		Gymnothorax kidako	AP002976
		Rhinomuraena quaesita	AP010844
	Synaphobranchidae	*Ilyophis brunneus*	AP010848
		Simenchelys parasitica	AP010849
		Synaphobranchus kaupii	AP002977
	Ophichthidae	*Myrichthys maculosus*	AP010862
	Colocongridae	*Coloconger cadenati*	AP010863
	Derichthyidae	*Derichthys serpentinus*	AP010851
		Nessorhamphus ingolfianus	AP010850
	Muraenesocidae	*Cynoponticus ferox*	AP010853
		Muraenesox bagio	AP010852
	Nemichthyidae	*Avocettina infans*	AP010855
		Labichthys carinatus	AP010856
		Nemichthys scolopaceus	AP010854
	Congridae	*Heteroconger hassi*	AP010859
		Leptocephalus sp. 'type II larva' (Smith, 1989)	AP010868
		Paraconger notialis	AP010860
	Nettastomatidae	*Facciolella oxyrhyncha*	AP010866

		Hoplunnis punctata	AP010865
		Nettastoma parviceps	AP010864
	Serrivomeridae	*Serrivomer beanii*	AP010857
		Serrivomer sector	AP007250
Clupeiformes	Denticipitidae	*Denticeps clupeoides*	AP007276
	Pristigasteridae	*Pellona flavipinnis*	AP009619
	Engraulidae	*Amazonsprattus scintilla*	AP009617
		Anchoviella sp. LBP 2297	AP011557
		Coilia lindmani	AP011558
		Coilia nasus	AP009135
		Coilia reynaldi	AP011559
		Engraulis encrasicolus	AP009137
		Engraulis japonicus	AB040676
		Lycengraulis grossidens	AP011563
		Lycothrissa crocodilus	AP011562
		Thryssa baelama	AP009616
	Chirocentridae	*Chirocentrus dorab*	AP006229
	Clupeidae	*Alosa alosa*	AP009131
		Alosa pseudoharengus	AP009132
		Alosa sapidissima	HQ331537
		Brevoortia tyrannus	AP009618
		Clupea harengus	AP009133
		Clupea pallasii	AP009134
		Clupeichthys aesarnensis	AP011584
		Clupeichthys goniognathus	AP011589
		Clupeichthys perakensis	AP011585
		Clupeoides borneensis	AP011586
		Clupeoides sp. Chao Phraya	AP011587
		Clupeonella cultriventris	AP009615
		Dorosoma cepedianum	DQ536426
		Dorosoma petenense	AP009136
		Ehirava fluviatilis	AP011588
		Escualosa thoracata	AP011601
		Ethmalosa fimbriata	AP009138
		Ethmidium maculatum	AP011602
		Etrumeus micropus	AP009139
		Gudusia chapra	AP011603
		Harengula jaguana	AP011592
		Hyperlophus vittatus	AP011593
		Ilisha africana	AP009140
		Ilisha elongata	AP009141
		Jenkinsia lamprotaenia	AP006230
		Konosirus punctatus	AP011612
		Microthrissa congica	AP011598
		Microthrissa royauxi	AP011596
		Myleus sp. NM-2010	AP011997
		Nematalosa japonica	AP009142
		Odaxothrissa losera	AP011595
		Odaxothrissa vittata	AP009231
		Pellonula leonensis	AP009232
		Potamalosa richmondia	AP011594
		Potamothrissa acutirostris	AP011597
		Potamothrissa obtusirostris	AP011599
		Sardina pilchardus	AP009233
		Sardinella albella	AP011605
		Sardinella maderensis	AP009143
		Sardinops melanostictus	AB032554

		Spratelloides delicatulus	AP009144
		Spratelloides gracilis	AP009145
		Sprattus antipodum	AP011608
		Sprattus muelleri	AP011607
		Sprattus sprattus	AP009234
		Sundasalanx mekongensis	AP006232
		Sundasalanx praecox	AP011590
		Sundasalanx sp. Chao Phraya	AP011591
		Tenualosa ilisha	AP011610
		Tenualosa thibaudeaui	AP011604
		Tenualosa toli	AP011600
Gonorynchiformes	Chanidae	*Chanos chanos*	AB054133
	Gonorynchidae	*Gonorynchus abbreviatus*	AP009402
		Gonorynchus greyi	AB054134
	Kneriidae	*Cromeria nilotica*	AP007275
		Grasseichthys gabonensis	AP007277
		Kneria sp. SL-2004	AP007278
		Parakneria cameronensis	AP007279
		Phractolaemus ansorgii	AP007280
Cypriniformes	Cyprinidae	*Acheilognathus intermedia*	EF483933
		Acheilognathus koreensis	EF483932
		Acheilognathus macropterus	EF483935
		Acheilognathus signifer	EF483930
		Acheilognathus somjinensis	FJ515921
		Acheilognathus typus	AB239602
		Acheilognathus yamatsutae	EF483936
		Alburnus alburnus	AB239593
		Aphyocypris chinensis	AB218688
		Barbonymus gonionotus	AB238966
		Barbus barbus	AB238965
		Barbus trimaculatus	AB239600
		Barilius bendelisis	AP011433
		Biwia zezera	AB250107
		Cabdio morar	AP011335
		Campostoma anomalum	DQ536421
		Carassius auratus	AB006953
		Carassius auratus auratus	AB111951
		Carassius auratus sp. 'Pingxiang'	HQ875340
		Carassius carassius	AY714387
		Carassius cuvieri	AB045144
		Carassius gibelio	GU170401
		Catla catla	JQ087872
		Chanodichthys mongolicus	AP009060
		Chondrostoma lemmingii	DQ536427
		Cirrhinus mrigala	JQ231112
		Coreoleuciscus splendidus	DQ347951
		Ctenopharyngodon idella	EU391390
		Culter alburnus	GU190362
		Cyprinella lutrensis	AB070206
		Cyprinella spiloptera	DQ536422
		Cyprinus carpio	X61010
		Cyprinus carpio carpio	JN105352
		Cyprinus carpio haematopterus	JN105354
		Cyprinus carpio wuyuanensis	JN105357
		Cyprinus carpio wuyuanensis x auratus	JN105356
		Cyprinus carpio xingguonensis	JN105353
		Danio dangila	AP011235

Danio rerio	AC024175
Distoechodon tumirostris	DQ026431
Esomus metallicus	AB239594
Gila conspersa	AP009315
Gila robusta	DQ536424
Gnathopogon elongatus	AB218687
Gobio gobio	AB239596
Gobiobotia brevibarba	FJ515919
Gobiobotia macrocephala	FJ515918
Gobiocypris rarus	JN116719
Gymnocypris przewalskii	AB239595
Hemibarbus barbus	AB070241
Hemibarbus labeo	DQ347953
Hemibarbus longirostris	DQ347952
Hemibarbus mylodon	DQ345787
Hemigrammocypris rasborella	AP011422
Hypophthalmichthys molitrix	EU315941
Hypophthalmichthys nobilis	EU343733
Ischikauia steenackeri	AB239601
Labeo bata	AP011198
Labeo batesii	AB238967
Labeo calbasu	JQ231113
Labeo rohita	JQ231111
Labeo senegalensis	AB238968
Leptobarbus hoevenii	AP011286
Luciosoma bleekeri	AP011399
Macrochirichthys macrochirus	AP011234
Megalobrama amblycephala	EU434747
Microdevario nana	AP011402
Microphysogobio koreensis	FJ515920
Mylocheilus caurinus	AP010779
Mylopharyngodon piceus	EU979305
Nicholsicypris normalis	AP011396
Notemigonus crysoleucas	AB127393
Notropis stramineus	DQ536429
Onychostoma lini	JQ343982
Opsariichthys bidens	DQ367044
Opsariichthys uncirostris	AB218897
Paralaubuca typus	AP011211
Pelecus cultratus	AB239597
Phenacobius mirabilis	DQ536431
Phoxinus eos	AP009151
Phoxinus percnurus	AP009149
Phoxinus percnurus mantschuricus	AP009061
Phoxinus percnurus sachalinensis	AP009150
Phoxinus steindachneri	AP009148
Procypris rabaudi	EU082030
Pseudaspius leptocephalus	AP009058
Pseudogobio esocinus	AP009310
Pseudopungtungia nigra	EU597300
Pseudopungtungia tenuicorpus	FJ515917
Pseudorasbora parva	JF802126
Pseudorasbora pumila	AB239599
Pungtungia herzi	AB239598
Puntius tetrazona	EU287909
Puntius ticto	AB238969
Raiamas guttatus	AP011222

		Raiamas senegalensis	AP010780
		Rasbora daniconius	AP011285
		Rasbora vaterifloris	AP011432
		Rhodeus ocellatus	DQ026430
		Rhodeus ocellatus kurumeus	AB070205
		Rhynchocypris lagowskii	AP009147
		Salmostoma bacaila	AP011223
		Sarcocheilichthys variegatus microoculus	AB054124
		Schizothorax biddulphi	JQ844133
		Sinocyclocheilus altishoulderus	FJ984568
		Sinocyclocheilus grahami	GQ148557
		Tanichthys albonubes	AP011397
		Tinca tinca	AB218686
		Tribolodon nakamurai	AB218896
		Trigonostigma espei	AP011449
		Xenocypris argentea	AP009059
		Xenocypris davidi	GQ289558
		Yaoshanicus arcus	AP011398
		Zacco sieboldii	AB218898
	Psilorhynchidae	*Psilorhynchus homaloptera*	DQ026436
		Psilorhynchus sucatio	AP011288
	Gyrinocheilidae	*Gyrinocheilus aymonieri*	AB242164
	Catostomidae	*Carpiodes carpio*	AY366087
		Catostomus commersonii	AB127394
		Cycleptus elongatus	AB126082
		Erimyzon oblongus	AP011228
		Hypentelium nigricans	AB242169
		Ictiobus bubalus	AP009316
		Minytrema melanops	DQ536432
		Moxostoma congestum	AP009317
		Moxostoma poecilurum	AB242167
		Myxocyprinus asiaticus	AY526869
		Xyrauchen texanus	EU265776
	Cobitidae	*Acantopsis choirorhynchos*	AB242161
		Chromobotia macracanthus	AB242163
		Cobitis choii	EU656112
		Cobitis sinensis	AY526868
		Cobitis striata	AB054125
		Cobitis takatsuensis	AP009306
		Koreocobitis naktongensis	HM535625
		Lefua echigonia	AB054126
		Leptobotia mantschurica	AB242170
		Misgurnus anguillicaudatus	DQ026434
		Misgurnus nikolskyi	AB242171
		Niwaella delicata	AP009308
		Pangio anguillaris	AB242168
	Balitoridae	*Barbatula toni*	AB242162
		Formosania lacustris	M91245
		Homaloptera leonardi	AB242165
		Schistura balteata	AB242172
		Sewellia lineolata	AP011292
		Sinogastromyzon puliensis	FJ605359
		Triplophysa stoliczkai	JQ663847
		Vaillantella maassi	AB242173
Characiformes	Distichodontidae	*Distichodus sexfasciatus*	AB070242
	Citharinidae	*Citharinus congicus*	AP011985
		Ichthyborus sp. NM-2010	AP011993

	Parodontidae	*Apareiodon affinis*	AP011998
	Chilodontidae	*Chilodus punctatus*	AP011984
	Hemiodontidae	*Hemiodopsis gracilis*	AP011990
	Alestiidae	*Chalceus macrolepidotus*	AB054130
		Micralestes sp. NM-2010	AP011996
		Phenacogrammus interruptus	AB054129
	Characidae	*Pygocentrus nattereri*	AP012000
	Acestrorhynchidae	*Acestrorhynchus* sp. NM-2010	AP011981
	Cynodontidae	*Hydrolycus scomberoides*	AP011989
	Lebiasinidae	*Lebiasina astrigata*	AP011995
	Hepsetidae	*Hepsetus odoe*	AP011991
Siluriformes	Diplomystidae	*Diplomystes nahuelbutaensis*	AP012011
	Amphylidae	*Amphilius* sp. NM-2010	AP012002
	Callichthyidae	*Corydoras rabauti*	AB054128
	Loricariidae	*Pterygoplichthys disjunctivus*	AP012021
	Amblycipitidae	*Liobagrus obesus*	DQ321752
	Aspredinidae	*Bunocephalus coracoideus*	AP012006
	Cranoglanididae	*Cranoglanis bouderius*	AY898626
		Pseudobagrus brevicorpus	HM355585
		Pseudobagrus tokiensis	AB054127
	Ictaluridae	*Ictalurus punctatus*	AF482987
	Mochokidae	*Synodontis schoutedeni*	AP012023
	Doradidae	*Amblydoras gonzalezi*	AP012001
	Auchenipteridae	*Centromochlus perugiae*	AP012024
		Tetranematichthys quadrifilis	AP012025
	Siluridae	*Silurus asotus*	AP012022
		Silurus glanis	AM398435
		Silurus lanzhouensis	JF895472
		Silurus meridionalis	HM746661
	Malapteruridae	*Malapterurus electricus*	AP012016
	Auchenoglanididae	*Auchenoglanis occidentalis*	AP012005
	Clariidae	*Clarias* sp. NM-2010	AP012010
	Heteropneustidae	*Heteropneustes fossilis*	AP012013
	Schilbeidae	*Pareutropius debauwi*	AP012017
	Pangasiidae	*Pangasianodon gigas*	AY762971
		Pangasius larnaudii	KC846907
	Bagridae	*Leiocassis longirostris*	GU596454
		Pelteobagrus vachellii	HM746660
		Tachysurus fulvidraco	KC287172
		Tachysurus nitidus	KC822643
	Pimelodidae	*Pimelodus pictus*	AP012019
Gymnotiformes	Rhamphichthyidae	*Gymnorhamphichthys* sp. NM-2010	AP011980
	Hypopomidae	*Brachyhypopomus pinnicaudatus*	AP011570
	Sternopygidae	*Eigenmannia* sp. CBM-ZF-10620	AB054131
	Apteronotidae	*Apteronotus albifrons*	AB054132
Argentiniformes	Argentinidae	*Glossanodon semifasciatus*	AP004105
	Opisthoproctidae	*Opisthoproctus soleatus*	AP004110
	Microstomatidae	*Nansenia ardesiaca*	AP004106
	Platytroctidae	*Maulisia mauli*	AP009404
		Platytroctes apus	AP004107
	Bathylaconidae	*Herwigia kreffti*	AP009582
	Alepocephalidae	*Alepocephalus agassizii*	AP009570
		Alepocephalus australis	AP009571
		Alepocephalus bairdii	AP009572
		Alepocephalus bicolor	AP009399
		Alepocephalus productus	AP009574
		Alepocephalus umbriceps	AP009575

		Bajacalifornia megalops	AP009578
		Bathyprion danae	AP009400
		Bathytroctes breviceps	AP009576
		Bathytroctes macrolepis	AP009577
		Bathytroctes michaelsarsi	AP009579
		Bathytroctes microlepis	AP009401
		Conocara macropterum	AP009580
		Conocara murrayi	AP009581
		Leptochilichthys agassizii	AP009403
		Leptoderma lubricum	AP009583
		Leptoderma macrophthalmum	AP011500
		Leptoderma retropinna	AP009584
		Narcetes erimelas	AP009405
		Narcetes stomias	AP009585
		Talismania bifurcata	AP009587
Osmeriformes	Osmeridae	*Hypomesus nipponensis*	HM106489
		Mallotus villosus	HM106491
		Osmerus mordax	HM106493
		Plecoglossus altivelis	AB047553
		Salangichthys microdon	AP004109
		Salanx ariakensis	AP006231
	Retropinnidae	*Retropinna retropinna*	AP004108
	Galaxiidae	*Galaxias gollumoides*	HM106487
		Galaxias maculatus	AP004104
		Galaxias sp. 'southern'	HM106488
Salmoniformes	Salmonidae	*Coregonus lavaretus*	JQ390060
		Hucho bleekeri	HM804473
		Hucho taimen	HQ897271
		Oncorhynchus clarkii henshawi	AY886762
		Oncorhynchus gorbuscha	EF455489
		Oncorhynchus keta	AP010773
		Oncorhynchus kisutch	EF126369
		Oncorhynchus masou 'Biwa'	EF105342
		Oncorhynchus masou formosanus	DQ858456
		Oncorhynchus masou ishikawae	DQ864464
		Oncorhynchus masou masou	DQ864465
		Oncorhynchus mykiss	L29771
		Oncorhynchus nerka	EF055889
		Oncorhynchus tshawytscha	AF392054
		Salmo salar	U12143
		Salmo trutta trutta	JQ390057
		Salvelinus alpinus	AF154851
		Salvelinus fontinalis	AF154850
		Thymallus arcticus	FJ872559
		Thymallus thymallus	FJ853655
Esociformes	Esocidae	*Esox lucius*	AP004103
Stomiiformes	Diplophidae	*Diplophos taenia*	AB034825
Ateleopodiformes	Ateleopodidae	*Ateleopus japonicus*	AP002916
		Ijimaia dofleini	AP002917
Aulopiformes	Aulopidae	*Aulopus japonicus*	AB047821
	Synodontidae	*Harpadon microchir*	AP002919
		Saurida undosquamis	AP002920
		Synodus variegatus	AY524977
	Chlorophthalmidae	*Chlorophthalmus agassizi*	AP002918
Myctophiformes	Neoscopelidae	*Neoscopelus microchir*	AP002921
Lampriformes	Lampridae	*Lampris guttatus*	AP002924
	Stylephoridae	*Stylephorus chordatus*	AB280687

Polymixiiformes	Polymixiidae	*Polymixia japonica*	AB034826
		Polymixia lowei	AP002927
Percopsiformes	Aphredoderidae	*Aphredoderus sayanus*	AP004403
Gadiformes	Macrouridae	*Coelorinchus kishinouyei*	AP002929
		Ventrifossa garmani	AP008991
	Moridae	*Physiculus japonicus*	AP004409
	Merlucciidae	*Merluccius merluccius*	FR751402
	Gadidae	*Arctogadus glacialis*	AM919429
		Gadus chalcogrammus	AB094061
		Gadus morhua	X99772
		Gadus ogac	DQ356941
		Lota lota	AP004412
		Melanogrammus aeglefinus	DQ020497
		Merlangius merlangus	DQ020496
		Micromesistius poutassou	FR751401
		Pollachius pollachius	FR751400
		Pollachius virens	FR751399
Ophidiiformes	Ophidiidae	*Bassozetus zenkevitchi*	AP004405
		Lamprogrammus niger	AP004410
		Sirembo imberbis	AP004406
	Bythitidae	*Cataetyx rubrirostris*	AP004407
		Diplacanthopoma brachysoma	AP004408
Lophiiformes	Lophiidae	*Lophiodes caulinaris*	AB282826
		Lophiomus setigerus	AP004413
		Sladenia gardineri	AB282827
	Chaunacidae	*Chaunax abei*	AP004415
		Chaunax pictus	AB282833
		Chaunax tosaensis	AP004416
	Ogcocephalidae	*Coelophrys brevicaudata*	AB282834
	Caulophrynidae	*Caulophryne pelagica*	AB282836
	Neoceratiidae	*Neoceratias spinifer*	AB282837
	Himantolophidae	*Himantolophus albinares*	AB282839
		Himantolophus groenlandicus	AB282840
	Diceratiidae	*Bufoceratias thele*	AB282841
		Diceratias pileatus	AB282842
Mugiliformes	Mugilidae	*Agonostomus monticola*	JF911702
		Aldrichetta forsteri	JF911703
		Chaenomugil proboscideus	JF911705
		Chelon labrosus	JF911706
		Crenimugil crenilabis	JF911707
		Liza affinis	JF911709
		Mugil cephalus	AP002930
		Mugil curema	JF911710
		Myxus capensis	JF911711
		Oedalechilus labeo	JF911712
		Rhinomugil nasutus	JF911714
		Sicamugil cascasia	JF911715
Atheriniformes	Atherinopsidae	*Menidia menidia*	AB370893
		Odontesthes sp. Odsp-001	AB370894
	Notocheiridae	*Iso hawaiiensis*	AB373006
	Melanotaeniidae	*Melanotaenia lacustris*	AP004419
	Atherinidae	*Hypoatherina tsurugae*	AP004420
Beloniformes	Adrianichthyidae	*Oryzias dancena*	AB498069
		Oryzias javanicus	AB498067
		Oryzias latipes	AP004421
		Oryzias luzonensis	AB498064
		Oryzias minutillus	AB498068

		Oryzias sarasinorum	AB370891
		Oryzias sinensis	GU013788
	Exocoetidae	*Cypselurus hiraii*	AB182653
		Exocoetus volitans	AP002933
	Belonidae	*Ablennes hians*	AB373007
	Scomberesocidae	*Cololabis saira*	AP002932
Cyprinodontiformes	Aplocheilidae	*Aplocheilus panchax*	AB373005
		Nothobranchius furzeri	EU650204
	Fundulidae	*Fundulus diaphanus*	FJ445394
		Fundulus grandis	FJ445396
		Fundulus heteroclitus	FJ445402
		Fundulus olivaceus	AP006776
	Cyprinodontidae	*Cyprinodon rubrofluviatilis*	EF442803
		Jordanella floridae	AP006778
	Poeciliidae	*Gambusia affinis*	AP004422
		Xiphophorus hellerii	FJ226476
		Xiphophorus maculatus	AP005982
Stephanoberyciformes	Rondeletiidae	*Rondeletia loricata*	AP002937
	Barbourisiidae	*Barbourisia rufa*	AP010879
	Cetomimidae	*Cetomimus* sp. AMS I34481001	AP010881
		Cetostoma regani	AP004423
		Danacetichthys galathenus	AP002936
		Gyrinomimus myersi	AP010884
		Gyrinomimus sp. UW 113191	AP010883
		Procetichthys kreffti	AP010880
	Mirapinnidae	*Eutaeniophorus festivus*	AP010885
		Eutaeniophorus sp. 033-Miya	AP004424
		Parataeniophorus gulosus	AP010886
	Megalomycteridae	*Ataxolepis apus*	AP010887
Beryciformes	Anoplogastridae	*Anoplogaster cornuta*	AP004425
	Anomalopidae	*Anomalops katoptron*	AP004428
	Monocentridae	*Monocentris japonicus*	AP004429
	Trachichthyidae	*Hoplostethus japonicus*	AP002938
	Berycidae	*Beryx decadactylus*	AP004430
		Beryx mollis	DQ993168
		Beryx splendens	AP002939
	Holocentridae	*Myripristis berndti*	AP002940
		Ostichthys japonicus	AP004431
		Sargocentron rubrum	AP004432
Zeiformes	Oreosomatidae	*Allocyttus niger*	AP004435
		Neocyttus rhomboidalis	AP004436
	Parazenidae	*Parazen pacificus*	AP004433
	Zenionidae	*Zenion japonicum*	AP004434
	Zeidae	*Zenopsis nebulosus*	AP002942
		Zeus faber	AP002941
Gasterosteiformes	Hypoptychidae	*Hypoptychus dybowskii*	AP004437
	Aulorhynchidae	*Aulichthys japonicus*	AB445127
		Aulorhynchus flavidus	AP009196
	Gasterosteidae	*Apeltes quadracus*	AB445126
		Culaea inconstans	AB445125
		Gasterosteus aculeatus	AP002944
		Gasterosteus wheatlandi	AB445129
		Pungitius kaibarae	EU332749
		Pungitius pungitius	AB445130
		Pungitius sinensis	EU332748
		Spinachia spinachia	AB445128
	Indostomidae	*Indostomus paradoxus*	AP004438

	Pegasidae	*Eurypegasus draconis*	AP005983
		Pegasus volitans	AP005984
	Solenostomidae	*Solenostomus cyanopterus*	AB277725
	Syngnathidae	*Hippocampus kuda*	AP005985
		Microphis brachyurus	AP005986
	Fistulariidae	*Fistularia commersonii*	AP005987
	Macroramphosidae	*Macroramphosus scolopax*	AP005988
	Centriscidae	*Aeoliscus strigatus*	AP009198
Synbranchiformes	Synbranchidae	*Monopterus albus*	AP002945
	Mastacembelidae	*Mastacembelus favus*	AP002946
Scorpaeniformes	Dactylopteridae	*Dactyloptena peterseni*	AP002947
		Dactyloptena tiltoni	AP004440
	Scorpaenidae	*Helicolenus hilgendorfi*	AP002948
		Sebastes schlegelii	AY491978
		Sebastiscus marmoratus	GU452728
	Peristediidae	*Scalicus amiscus*	AP004441
	Cottidae	*Cottus hangiongensis*	EU332751
		Cottus poecilopus	EU332750
		Cottus reinii	AP004442
	Cyclopteridae	*Aptocyclus ventricosus*	AP004443
Perciformes	Latidae	*Lates calcarifer*	DQ010541
	Moronidae	*Coreoperca kawamebari*	AP005990
		Lateolabrax japonicus	JQ860109
		Morone saxatilis	HM447585
		Siniperca chuatsi	JF972568
		Siniperca knerii	JN378751
		Siniperca scherzeri	JN084101
	Percichthyidae	*Nannoperca australis*	JF519732
		Nannoperca obscura	JF519733
	Acropomatidae	*Doederleinia berycoides*	AP009181
	Serranidae	*Anyperodon leucogrammicus*	GQ131336
		Epinephelus akaara	EU043377
		Epinephelus bruneus	FJ594964
		Epinephelus coioides	EU043376
		Epinephelus lanceolatus	FJ472837
		Epinephelus moara	JQ518290
		Hyporthodus septemfasciatus	FJ594966
		Plectropomus leopardus	DQ101270
	Pseudochromidae	*Labracinus cyclophthalmus*	AP009125
	Opistognathidae	*Opistognathus jacksoniensis*	JF911713
	Centrarchidae	*Lepomis macrochirus*	JN389795
		Micropterus dolomieu	AB378749
		Micropterus floridanus	HQ391897
		Micropterus salmoides	DQ536425
		Micropterus salmoides salmoides	HQ391896
	Percidae	*Etheostoma radiosum*	AY341348
		Percina macrolepida	DQ536430
	Sillaginidae	*Sillago sihama*	JQ048935
	Malacanthidae	*Branchiostegus albus*	EU861053
		Branchiostegus argentatus	EU861054
		Branchiostegus japonicus	EU861052
	Carangidae	*Carangoides armatus*	AP004444
		Caranx melampygus	AP004445
		Seriola dumerili	AB517558
		Seriola lalandi	AB517557
		Seriola quinqueradiata	AB517556
		Trachurus japonicus	AP003091

	Trachurus trachurus	AB108498
Emmelichthyidae	*Emmelichthys struhsakeri*	AP004446
Lutjanidae	*Lutjanus argentimaculatus*	JN182927
	Lutjanus bengalensis	FJ171339
	Lutjanus kasmira	FJ416614
	Lutjanus malabaricus	FJ824741
	Lutjanus rivulatus	AP006000
	Lutjanus russellii	EF514208
	Lutjanus sebae	FJ824742
	Pterocaesio tile	AP004447
Haemulidae	*Diagramma picta*	AP009167
	Hapalogenys nigripinnis	HM754620
	Parapristipoma trilineatum	AP009168
Lethrinidae	*Monotaxis grandoculis*	AP009166
Sparidae	*Acanthopagrus latus*	EF506764
	Pagellus bogaraveo	AB305023
	Pagrus auriga	AB124801
	Pagrus major	AP002949
	Parargyrops edita	EF107158
	Spicara maena	AP009164
Sciaenidae	*Argyrosomus japonicus*	JQ728563
	Chrysochir aureus	JQ692068
	Collichthys lucidus	HM447239
	Collichthys niveatus	HM219223
	Dendrophysa russelii	JQ728562
	Larimichthys crocea	EU339149
	Larimichthys polyactis	FJ618559
	Miichthys miiuy	HM447240
	Nibea albiflora	HQ890947
	Pennahia argentata	HQ890946
	Sciaenops ocellatus	JQ286004
Monodactylidae	*Monodactylus argenteus*	AP009169
Toxotidae	*Toxotes chatareus*	AP006806
Arripidae	*Arripis trutta*	AP006810
Kyphosidae	*Girella punctata*	AP011060
	Kyphosus cinerascens	AP011061
	Labracoglossa argentiventris	AP011062
	Microcanthus strigatus	AP006009
	Oplegnathus fasciatus	DQ872160
	Oplegnathus punctatus	AP011066
	Scorpis lineolata	AP011063
Chaetodontidae	*Chaetodon auripes*	AP006004
	Heniochus diphreutes	AP006005
Pomacanthidae	*Centropyge loricula*	AP006006
	Chaetodontoplus septentrionalis	AP006007
Enoplosidae	*Enoplosus armatus*	AP006008
Pentacerotidae	*Histiopterus typus*	AP006807
Terapontidae	*Rhynchopelates oxyrhynchus*	AP011064
Elassomatidae	*Elassoma evergladei*	AP002950
	Elassoma zonatum	AP006813
Cichlidae	*Astronotus ocellatus*	AP009127
	Etroplus maculatus	AP009505
	Hypselecara temporalis	AP009506
	Neolamprologus brichardi	AP006014
	Oreochromis aureus	GU370125
	Oreochromis mossambicus	AY597335
	Oreochromis niloticus	GU238433

	Oreochromis sp. 'red tilapia'	HM067614
	Oreochromis sp. KM-2006	AP009126
	Paratilapia polleni	AP009508
	Paretroplus maculatus	AP009504
	Ptychochromoides katria	AP009507
	Sarotherodon melanotheron	JF894132
	Tropheus duboisi	AP006015
	Tylochromis polylepis	AP009509
Embiotocidae	*Cymatogaster aggregata*	AP009128
	Ditrema temminckii	AP009129
Pomacentridae	*Abudefduf vaigiensis*	AP006016
	Amphiprion bicinctus	JQ030887
	Amphiprion ocellaris	AP006017
Labridae	*Halichoeres melanurus*	AP006018
	Halichoeres tenuispinis	EU082205
	Halichoeres trimaculatus	EU087704
	Parajulis poecilepterus	EF192032
	Pseudolabrus eoethinus	EU560728
	Pseudolabrus sieboldi	AP006019
	Pteragogus flagellifer	EF409976
Odacidae	*Odax cyanomelas*	AP009130
Scaridae	*Chlorurus sordidus*	AP006567
	Scarus forsteni	FJ619271
	Scarus ghobban	FJ449707
	Scarus rubroviolaceus	FJ227899
	Scarus schlegeli	FJ595020
Pholidae	*Pholis crassispina*	AP004449
Anarhichadidae	*Anarhichas lupus*	EF427916
Nototheniidae	*Notothenia coriiceps*	JF933906
	Pleuragramma antarctica	JF933905
Channichthyidae	*Chaenocephalus aceratus*	JF933907
	Chionodraco myersi	DQ526430
Trichodontidae	*Arctoscopus japonicus*	AP003090
Blenniidae	*Petroscirtes breviceps*	AP004450
	Salarias fasciatus	AP004451
Gobiesocidae	*Arcos* sp. KU-149	AP004452
Rhyacichthyidae	*Rhyacichthys aspro*	AP004454
Eleotridae	*Bostrychus sinensis*	JQ665462
	Eleotris acanthopoma	AP004455
Gobiidae	*Acanthogobius hasta*	AY486321
	Acentrogobius pflaumii	KC196074
	Boleophthalmus pectinirostris	JN631352
	Gillichthys mirabilis	FJ211845
	Gillichthys seta	FJ211846
	Glossogobius olivaceus	JQ001860
	Gymnogobius petschiliensis	AY525784
	Oxuderces dentatus	JN831381
	Scartelaos histophorus	JQ654459
	Stiphodon alcedo	AB613000
	Tridentiger bifasciatus	JN244650
	Trypauchen vagina	JQ027694
Ephippidae	*Platax orbicularis*	AP006825
Siganidae	*Siganus fuscescens*	EF025185
	Siganus unimaculatus	AP006031
Luvaridae	*Luvarus imperialis*	AP009161
Zanclidae	*Zanclus cornutus*	AP009162
Acanthuridae	*Acanthurus leucosternon*	EU136032

		Acanthurus lineatus	EU273284
		Naso lopezi	AP009163
		Zebrasoma flavescens	AP006032
	Trichiuridae	*Trichiurus japonicus*	EU339148
	Scombridae	*Auxis rochei*	AB103467
		Auxis thazard	AB105447
		Euthynnus alletteratus	AB099716
		Katsuwonus pelamis	AB101290
		Rastrelliger brachysoma	EU555283
		Scomber australasicus	AB488407
		Scomber colias	AB488406
		Scomber japonicus	AB488405
		Scomber scombrus	AB120717
		Scomberomorus cavalla	DQ536428
		Scomberomorus niphonius	GU109281
		Thunnus alalunga	AB101291
		Thunnus albacares	GU256528
		Thunnus maccoyii	GU256523
		Thunnus obesus	GU256525
		Thunnus orientalis	AB185022
		Thunnus thynnus	GU256522
		Thunnus thynnus thynnus	AY302574
	Xiphiidae	*Xiphias gladius*	AB470301
	Istiophoridae	*Makaira indica*	AB470305
		Istiophorus platypterus	AB470306
		Kajikia audax	AB470302
		Makaira mazara	AB470304
		Tetrapturus angustirostris	AB470303
	Centrolophidae	*Hyperoglyphe japonica*	AB752307
	Nomeidae	*Cubiceps pauciradiatus*	AP006038
		Psenes cyanophrys	AP011067
	Stromateidae	*Pampus* sp. LY-2009	EU357803
	Channidae	*Channa argus*	GU937112
	Caproidae	*Antigonia capros*	AP002943
		Capros aper	AP009159
Pleuronectiformes	Paralichthyidae	*Paralichthys olivaceus*	AB028664
	Pleuronectidae	*Hippoglossus hippoglossus*	AM749122
		Hippoglossus stenolepis	AM749126
		Kareius bicoloratus	AP002951
		Platichthys stellatus	EF424428
		Reinhardtius hippoglossoides	AM749130
		Verasper moseri	EF025506
		Verasper variegatus	DQ403797
	Soleidae	*Solea senegalensis*	KF142459
	Cynoglossidae	*Cynoglossus abbreviatus*	GQ380410
Tetraodontiformes	Triacanthodidae	*Macrorhamphosodes uradoi*	AP009171
		Triacanthodes anomalus	AP009172
	Triacanthidae	*Triacanthus biaculeatus*	AP009174
		Trixiphichthys weberi	AP009173
	Balistidae	*Abalistes stellaris*	AP009202
		Balistapus undulatus	AP009203
		Balistes vetula	AP009204
		Balistoides conspicillum	AP009205
		Canthidermis maculata	AP009206
		Melichthys vidua	AP009207
		Odonus niger	AP009208
		Pseudobalistes flavimarginatus	AP009209

	Rhinecanthus aculeatus	AP009210
	Sufflamen fraenatum	AP004456
	Xanthichthys auromarginatus	AP009211
	Xenobalistes tumidipectoris	AP009182
Monacanthidae	*Acanthaluteres brownii*	AP009212
	Acreichthys tomentosus	AP009213
	Aluterus scriptus	AP009183
	Amanses scopas	AP009214
	Brachaluteres ulvarum	AP009215
	Cantherhines pardalis	AP009184
	Chaetodermis penicilligerus	AP009216
	Eubalichthys mosaicus	AP009217
	Meuschenia hippocrepis	AP009218
	Monacanthus chinensis	AP009219
	Nelusetta ayraudi	AP009220
	Paraluteres prionurus	AP009222
	Paramonacanthus choirocephalus	AP009223
	Pervagor janthinosoma	AP009224
	Pseudomonacanthus peroni	AP009225
	Rudarius ercodes	AP009227
	Scobinichthys granulatus	AP009228
	Stephanolepis cirrhifer	AP002952
	Thamnaconus modestus	AP009185
Ostraciidae	*Anoplocapros lenticularis*	AP009186
	Kentrocapros aculeatus	AP009175
	Lactoria diaphana	AP009187
	Ostracion immaculatus	AP009176
Triodontidae	*Triodon macropterus*	AP009170
Tetraodontidae	*Arothron firmamentum*	AP006742
	Arothron hispidus	AP011930
	Arothron manilensis	AP011929
	Canthigaster coronata	AP006743
	Canthigaster jactator	AP011911
	Canthigaster rivulata	AP006744
	Canthigaster valentini	AP006744
	Carinotetraodon lorteti	AP011918
	Carinotetraodon salivator	AP011919
	Chelonodon pleurospilus	AP011928
	Colomesus asellus	AP011909
	Colomesus psittacus	AP011910
	Lagocephalus laevigatus	AP011934
	Lagocephalus lagocephalus	AP011933
	Lagocephalus lunaris	GQ461750
	Lagocephalus sceleratus	AP011932
	Lagocephalus wheeleri	AP009538
	Leiodon cutcutia	AP011924
	Marilyna darwinii	AP011937
	Monotrete cochinchinensis	KF667490
	Omegophora armilla	AP011936
	Pelagocephalus marki	AP011938
	Polyspina piosae	AP011913
	Sphoeroides annulatus	AP011915
	Sphoeroides pachygaster	AP006745
	Sphoeroides parvus	AP011914
	Sphoeroides testudineus	AP011916
	Takifugu chinensis	AP009534
	Takifugu chrysops	AP009525

		Takifugu exascurus	AP009540
		Takifugu fasciatus	GQ409967
		Takifugu niphobles	AP009526
		Takifugu oblongus	AP009535
		Takifugu obscurus	AP009527
		Takifugu ocellatus	AP009536
		Takifugu pardalis	AP009528
		Takifugu poecilonotus	AP009539
		Takifugu porphyreus	AP009529
		Takifugu rubripes	AJ421455
		Takifugu snyderi	AP009531
		Takifugu stictonotus	AP009530
		Takifugu vermicularis	AP009532
		Takifugu xanthopterus	AP009533
		Tetractenos glaber	AP011935
		Tetraodon biocellatus	AP011921
		Tetraodon mbu	AP011923
		Tetraodon miurus	AP011922
		Tetraodon nigroviridis	DQ019313
		Tetraodon palembangensis	AP011920
		Torquigener brevipennis	AP009537
		Torquigener hypselogeneion	AP011927
		Torquigener pleurogramma	AP011926
		Tylerius spinosissimus	AP011939
	Diodontidae	*Chilomycterus reticulatus*	AP009188
		Diodon holocanthus	AP009177
	Molidae	*Masturus lanceolatus*	AP006239
		Mola mola	AP006238
		Ranzania laevis	AP006047
Ceratodontiformes	Ceratodontidae	*Neoceratodus forsteri*	AF302933
	Lepidosirenidae	*Lepidosiren paradoxa*	AF302934

Table S2.
A list of fish species used for designing universal primers specifically designed for elasmobranchs (MiFish-E) including 160 species placed in 12 orders, 39 families and 77 genera.

Order	Family	Species	Accession No.
Heterodontiformes	Heterodontidae	*Heterodontus francisci*	AJ310141
		Heterodontus zebra	KC845548
Orectolobiformes	Orectolobidae	*Orectolobus japonicus*	KF111729
	Hemiscylliidae	*Chiloscyllium griseum*	JQ434458
		Chiloscyllium plagiosum	JX162601
		Chiloscyllium punctatum	JQ082337
	Ginglymostomatidae	*Ginglymostoma cirratum*	AY147890
	Rhincodontidae	*Rhincodon typus*	KC633221
Lamniformes	Odontaspididae	*Carcharias taurus*	KF569943
	Mitsukurinidae	*Mitsukurina owstoni*	EU528659
	Megachasmidae	*Megachasma pelagios*	KC702506
	Alopidae	*Alopias pelagicus*	KF412639
		Alopias superciliosus	KC757415
		Alopias vulpinus	AY147892
	Cetorhinidae	*Cetorhinus maximus*	KF597303
	Lamnidae	*Carcharodon carcharias*	AY147893
		Isurus oxyrinchus	KF361861
		Isurus paucus	KJ616742
		Lamna ditropis	KF962053
		Lamna nasus	AY147896
Carcharhiniformes	Scyliorhinidae	*Apristurus longicephalus*	GU130599
	Pseudotriakidae	*Pseudotriakis microdon*	AB560493
	Triakidae	*Mustelus griseus*	KF889325
		Mustelus manazo	AB015962
	Carcharhinidae	*Carcharhinus acronotus*	KF728380
		Carcharhinus amblyrhynchoides	KF956523
		Carcharhinus falciformis	KF801102
		Carcharhinus leucas	KF646785
		Carcharhinus melanopterus	KJ720818
		Carcharhinus obscurus	KC470543
		Carcharhinus sorrah	KF612341
		Galeocerdo cuvier	KF111728
		Glyphis garricki	KF646786
		Glyphis glyphis	KF006312
		Prionace glauca	KF356249
		Scoliodon macrorhynchos	JQ693102
	Sphyrnidae	*Sphyrna lewini*	JX827259
		Sphyrna mokarran	AF448022
		Sphyrna zygaena	AF448023
Hexanchiformes	Chlamydoselachidae	*Chlamydoselachus anguineus*	AB560487
	Hexanchidae	*Heptranchias perlo*	AY147888
		Hexanchus griseus	AY147887
		Hexanchus nakamurai	AB560491
		Notorynchus cepedianus	AB560489
Squaliformes	Squalidae	*Cirrhigaleus australis*	KJ128289
		Squalus acanthia	Y18134
		Squalus megalops	GU130625
	Centrophoridae	*Centrophorus granulosus*	AY147884
		Centrophorus squamosus	GU130628
		Deania calcea	GU130626
	Etmopteridae	*Aculeola nigra*	GU130605

		Centroscyllium fabricii	GU130634
		Centroscyllium granulatum	GU130635
		Centroscyllium nigrum	GU130632
		Centroscyllium ritteri	GU130633
		Etmopterus baxteri	GU130661
		Etmopterus bigelowi	GU130617
		Etmopterus brachyurus	GU130643
		Etmopterus cf. *decacuspidatus*	GU130611
		Etmopterus cf. *granulosus*	GU130658
		Etmopterus cf. *molleri*	GU130612
		Etmopterus dianthus	GU130620
		Etmopterus dislineatus	GU130608
		Etmopterus fusus	GU130614
		Etmopterus gracilispinis	GU130651
		Etmopterus granulosus	GU130606
		Etmopterus lucifer	GU130609
		Etmopterus molleri	GU130637
		Etmopterus polli	GU130669
		Etmopterus princeps	GU130654
		Etmopterus pseudosqualiolus	GU130613
		Etmopterus pusillus	GU130615
		Etmopterus schultzi	GU130646
		Etmopterus sentosus	GU130647
		Etmopterus sheikoi	GU130607
		Etmopterus sp. B	GU130619
		Etmopterus spinax	GU130618
		Etmopterus unicolor	GU130666
		Etmopterus virens	GU130670
		Trigonognathus kabeyai	GU130629
	Somniosidae	*Centroscymnus owstoni*	GU130622
		Centroselachus crepidater	GU130621
		Proscymnodon plunketi	GU130623
		Scymnodon ringens	GU130624
		Somniosus microcephalus	GU130604
		Somniosus pacificus	AB560492
	Oxynotidae	*Oxynotus paradoxus*	GU130601
	Dalatiidae	*Dalatias licha*	GU130603
		Squaliolus aliae	GU130602
Squatiniformes	Squatinidae	*Squatina californica*	AY147886
		Squatina japonica	KJ619663
		Squatina nebulosa	AF448031
Pristiophoriformes	Pristiophoridae	*Pristiophorus japonicus*	AB721306
		Pristiophorus nudipinnis	AY147885
Torpediniformes	Torpedinidae	*Torpedo marmorata*	EF100173
		Torpedo tokionis	AF448026
	Narcinidae	*Narcine timlei*	AF448002
Pristiformes	Pristidae	*Anoxypristis cuspidata*	AF447988
		Pristis clavata	KF381507
Rajiformes	Rhinidae	*Rhina ancylostoma*	AF448013
	Rhynchobatidae	*Rhynchobatus djiddensis*	AF448020
	Rhinobatidae	*Rhinobatos cemiculus*	EF100174
		Rhinobatos formosensis	AF448014
		Rhinobatos hynnicephalus	AF448015
		Rhinobatos schlegelii	AF448016
	Rajidae	*Amblyraja hyperborea*	EF100184
		Dipturus batis	EF081271
		Dipturus kwangtungensis	KF318309

		Dipturus nidarosiensis	EF081266
		Dipturus oxyrinchus	EF081269
		Dipturus tengu	EF081265
		Hongeo koreana	KC914433
		Leucoraja erinacea	JQ034406
		Leucoraja fullonica	EF100179
		Leucoraja naevus	EF100181
		Malacoraja kreffti	EF081262
		Neoraja caerulea	EF100178
		Okamejei acutispina	EF100189
		Raja acutispina	AF448009
		Raja brachyura	EF081263
		Raja clavata	EF100186
		Raja fyllae	EF100182
		Raja kukujevi	EF100183
		Raja kwangtungensis	AF448010
		Raja macrocauda	AF448011
		Raja microocellata	EF081264
		Raja montagui	EF100188
		Raja polystigma	EF100185
		Raja porosa	AY525783
		Raja radiata	AF106038
		Raja rhina	KC914434
		Raja undulata	EF100187
		Rostroraja alba	EF081261
		Zearaja chilensis	KF648508
Myliobatiformes	Platyrhinidae	*Platyrhina sinensis*	AF448004
	Hexatrygonidae	*Plesiobatis daviesi*	AF448005
	Urolophidae	*Urobatis jamaicensis*	AY147898
	Dasyatidae	*Dasyatis akajei*	KC526959
		Dasyatis bennetti	KC196067
		Dasyatis kuhlii	AF447991
		Dasyatis sp. KCCL	AF447992
		Dasyatis thetidis	AF447993
		Dasyatis zugei	JX524174
		Himantura gerrardi	AF447996
		Himantura granulata	KF751650
		Himantura uarnak	AF447997
		Neotrygon kuhlii	KC992792
		Pastinachus atrus	HG942172
		Pteroplatytrygon violacea	KJ641617
		Taeniura lymma	AF448024
		Taeniura melanospilos	AF448025
		Taeniura meyeni	JX827260
		Urogymnus asperrimus	AF448027
	Gymnuridae	*Aetoplatea zonura*	AF447986
		Gymnura poecilura	KJ617038
	Myliobatidae	*Aetobatus flagellum*	KF482070
		Manta birostris	AF448000
		Mobula formosana	AF448001
		Mobula japanica	JX392983
		Rhinoptera javanica	AF448019

Table S3.
A list of fish species used for constructing the custom database for taxonomic assignment using BLAST including 648 sequences from 594 species placed in 36 orders, 162 families and 390 genera.

Order	Family	Species	Accession No.
Petromyzontiformes	Petromyzontidae	*Lethenteron reissneri*	AB969938
		Lethenteron reissneri	AB969939
Chimaeriformes	Rhinochimaeridae	*Rhinochimaera africana*	AB938083
Heterodontiformes	Heterodontidae	*Heterodontus japonicus*	AB938085
Orectolobiformes	Orectolobidae	*Orectolobus japonicus*	AB938086
	Rhincodontidae	*Rhincodon typus*	AB938087
Lamniformes	Odontaspididae	*Odontaspis ferox*	AB938088
	Megachasmidae	*Megachasma pelagios*	AB969940
	Alopiidae	*Alopias pelagicus*	AB938089
	Cetorhinidae	*Cetorhinus maximus*	AB974447
	Lamnidae	*Isurus oxyrinchus*	AB938090
Carcharhiniformes	Scyliorhinidae	*Scyliorhinus torazame*	AB974448
		Cephaloscyllium umbratile	AB974452
	Pentanchidae	*Apristurus macrorhynchus*	AB974449
		Galeus sauteri	AB974450
		Parmaturus pilosus	AB974451
	Proscylliidae	*Proscyllium venustum*	AB974453
	Triakidae	*Hemitriakis complicofasciata*	AB938091
		Mustelus griseus	AB974600
	Carcharhinidae	*Carcharhinus albimarginatus*	AB938093
		Carcharhinus brachyurus	AB938094
		Carcharhinus brevipinna	AB938095
		Carcharhinus dussumieri	AB938096
		Carcharhinus falciformis	AB938097
		Carcharhinus leucas	AB938098
		Carcharhinus limbatus	AB938099
		Carcharhinus obscurus	AB938100
		Carcharhinus plumbeus	AB938101
		Galeocerdo cuvier	AB938102
		Negaprion acutidens	AB969868
		Prionace glauca	AB938103
		Triaenodon obesus	AB938105
		Carcharodon carcharias	AB974599
	Sphyrnidae	*Sphyrna zygaena*	AB938104
		Sphyrna lewini	AB974454
Hexanchiformes	Chlamydoselachidae	*Chlamydoselachus anguineus*	AB974455
	Hexanchidae	*Hexanchus nakamurai*	AB938106
		Notorynchus cepedianus	AB938107
		Heptranchias perlo	AB974456
Squaliformes	Squalidae	*Squalus japonicus*	AB974457
		Squaliolus aliae	AB974458
	Centrophoridae	*Centrophorus atromarginatus*	AB969869
		Centrophorus tessellatus	AB974459
		Centrophorus moluccensis	AB974460
	Etmopteridae	*Etmopterus brachyurus*	AB938109
		Etmopterus lucifer	AB969870
		Etmopterus molleri	AB974461
		Etmopterus bigelowi	AB974462
	Somniosidae	*Zameus ichiharai*	AB970002
		Centroscymnus owstoni	AB974463
Squatiniformes	Squatinidae	*Squatina japonica*	AB938110

Torpediniformes	Narcinidae	*Narcine lingula*	AB970003
	Narkidae	*Narke japonica*	AB969941
Rajiformes	Rhinobatidae	*Rhina ancylostoma*	AB938113
		Rhynchobatus djiddensis	AB938114
	Rajidae	*Dipturus macrocauda*	AB938115
		Okamejei hollandi	AB969980
		Raja pulchra	AB938116
Myliobatiformes	Hexatrygonidae	*Hexatrygon bickelli*	AB969942
	Plesiobatidae	*Plesiobatis daviesi*	AB938117
	Urolophidae	*Urolophus aurantiacus*	AB938118
		Urolophus aurantiacus	AB938119
	Dasyatidae	*Dasyatis matsubarai*	AB938120
		Dasyatis ushiei	AB938121
		Himantura uarnak	AB938122
		Neotrygon kuhlii	AB974464
		Urogymnus asperrimus	AB938123
		Himantura uarnak	AB974465
		Dasyatis akajei	AB974466
		Pteroplatytrygon violacea	AB974467
		Dasyatis ushiei	AB974598
	Gymnuridae	*Gymnura japonica*	AB938124
		Gymnura japonica	AB974468
	Myliobatidae	*Aetobatus flagellum*	AB938125
		Aetobatus narinari	AB938126
		Manta alfredi	AB938127
		Mobula japonica	AB938128
		Mobula tarapacana	AB938129
		Mobula thurstoni	AB938130
		Mobula thurstoni	AB974469
		Myliobatis tobijei	AB938131
		Manta birostris	AB974470
Elopiformes	Elopidae	*Elops hawaiensis*	AB974471
Albuliformes	Albulidae	*Pterothrissus gissu*	AB974472
		Albula forsteri	AB974597
	Notacanthidae	*Notacanthus chemnitzii*	AB974473
Anguilliformes	Muraenidae	*Strophidon sathete*	AB969837
		Gymnothorax flavimarginatus	AB974474
		Enchelycore pardalis	AB969836
		Strophidon sathete	AB969838
		Gymnothorax niphostigmus	AB974475
	Ophichthidae	*Ophisurus macrorhynchos*	AB969839
		Ophichthus urolophus	AB974476
		Pisodonophis cancrivorus	AB974477
	Congridae	*Conger japonicus*	AB974480
		Macrocephenchelys brevirostris	AB974479
		Uroconger lepturus	AB974478
Clupeiformes	Clupeidae	*Sardinella melanura*	AB974481
Cypriniformes	Cyprinidae	*Tribolodon brandtii*	AP011418
		Carassius auratus grandoculis	AP011239
		Tanakia tanago	AP012526
		Squalidus gracilis gracilis	AP011393
		Rhynchocypris oxycephalus jouyi	AP011269
		Tribolodon sachalinensis	AP011270
		Phoxinus percnurus sachalinensis	AP011271
		Zacco platypus	AP012115
		Nipponocypris temminckii	AP012116
		Carassius cuvieri	AP011237

		Pseudogobio esocinus	AP011256
	Cobitidae	*Cobitis takatsuensis*	AP011290
		Misgurnus anguillicaudatus	AP011291
		Paramisgurnus dabryanus	AP012124
		Cobitis sp. BIWAE type B	AP011344
	Balitoridae	*Lefua nikkonis*	AP011300
Siluriformes	Plotosidae	*Plotosus japonicus*	AB974592
Argentiniformes	Bathylagidae	*Leuroglossus schmidti*	AB969999
		Nansenia sanrikuensis	AB970000
Stomiiformes	Phosichthyidae	*Polymetme elongatus*	AB969851
		Woodsia nonsuchae	AB969852
Ateleopodiformes	Ateleopodidae	*Guentherus katoi*	AB974482
Aulopiformes	Aulopidae	*Hime japonica*	AB969854
	Synodontidae	*Harpadon nehereus*	AB970004
		Saurida wanieso	AB938168
		Saurida wanieso	AB938169
		Synodus dermatogenys	AB969855
		Synodus doaki	AB969856
		Trachinocephalus myops	AB974483
	Chlorophthalmidae	*Chlorophthalmus nigromarginatus*	AB974484
Myctophiformes	Neoscopelidae	*Neoscopelus microchir*	AB969857
	Myctophidae	*Nannobrachium regale*	AB974485
		Benthosema pterotum	AB974486
		Diaphus suborbitalis	AB974487
Lampriformes	Veliferidae	*Metavelifer multiradiatus*	AB938171
		Velifer hypselopterus	AB969943
	Lampridae	*Lampris guttatus*	AB969858
	Lophotidae	*Lophotus capellei*	AB969859
	Trachipteridae	*Desmodema polystictum*	AB969965
		Trachipterus ishikawae	AB938163
		Zu cristatus	AB969860
	Regalecidae	*Regalecus russelii*	AB969944
		Regalecus russelii	AB938164
Gadiformes	Macrouridae	*Coelorinchus jordani*	AB969945
		Coelorinchus smithi	AB969862
		Coelorinchus hubbsi	AB969946
		Coryphaenoides marginatus	AB969947
		Coryphaenoides microps	AB970005
		Hymenocephalus gracilis	AB969863
		Hymenocephalus striatissimus	AB969864
		Ventrifossa garmani	AB969866
		Coelorinchus formosanus	AB974488
		Ventrifossa rhipidodorsalis	AB974489
		Coelorinchus anatirostris	AB974490
		Ventrifossa garmani	AB974491
		Ventrifossa garmani	AB974492
		Lucigadus nigromarginatus	AB974493
	Moridae	*Laemonema modestum*	AB969948
		Laemonema modestum	AB969949
		Lotella tosaensis	AB974494
		Gadella jordani	AB974495
		Physiculus japonicus	AB974496
	Lotidae	*Ciliata mustela*	AB969950
		Gaidropsarus argentatus	AB969951
Ophidiiformes	Parabrotulidae	*Parabrotula tanseimaru*	AB974497
Lophiiformes	Lophiidae	*Lophiomus setigerus*	AB969873
		Lophius litulon	AB974498

	Ogcocephalidae	*Halieutaea fumosa*	AB969875
		Halieutaea sp.	AB969876
		Malthopsis annulifera	AB969877
Atheriniformes	Atherinidae	*Iso flosmaris*	AB974499
Beloniformes	Exocoetidae	*Cypselurus doederleini*	AB974501
		Oxyporhamphus similis	AB972266
	Belonidae	*Tylosurus crocodilus crocodilus*	AB974500
Beryciformes	Anomalopidae	*Anomalops* sp.	AB974584
	Monocentridae	*Monocentris japonicus*	AB969881
		Monocentris japonicus	AB974588
	Trachichthyidae	*Paratrachichthys prosthemius*	AB974502
		Gephyroberyx japonicus	AB974503
		Hoplostethus japonicus	AB974504
		Hoplostethus sp. cf. *crassispinus*	AB974586
	Holocentridae	*Neoniphon sammara*	AB974505
		Neoniphon sammara	AB974506
		Ostichthys japonicus	AB969883
		Sargocentron punctatissimum	AB969884
		Sargocentron rubrum	AB974507
		Myripristis greenfieldi	AB974508
Zeiformes	Parazenidae	*Parazen pacificus*	AB974509
Gasterosteiformes	Fistulariidae	*Fistularia petimba*	AB974649
		Fistularia commersonii	AB974594
Scorpaeniformes	Dactylopteridae	*Dactyloptena peterseni*	AB969961
		Dactylopterus volitans	AB972264
	Scorpaenidae	*Apistus carinatus*	AB974510
		Pterois lunulata	AB969960
		Sebastes thompsoni	AB969889
		Sebastes trivittatus	AB969890
		Sebastes ventricosus	AB969891
		Sebastiscus marmoratus	AB969894
		Sebastiscus tertius	AB969892
		Setarches guentheri	AB969893
		Sebastolobus macrochir	AB972263
		Dendrochirus brachypterus	AB974512
		Sebastiscus albofasciatus	AB974513
		Apistus carinatus	AB974511
		Pterois volitans	AB974514
	Tetrarogidae	*Tetraroge barbata*	AB974687
	Synanceiidae	*Inimicus japonicus*	AB969887
	Triglidae	*Chelidonichthys spinosus*	AB974515
		Chelidonichthys spinosus	AB974516
		Pterygotrigla multipunctata	AB974517
		Lepidotrigla abyssalis	AB974518
		Lepidotrigla kishinouyei	AB974519
		Lepidotrigla sp.	AB974520
	Peristediidae	*Peristedion nierstraszi*	AB969897
		Peristedion orientale	AB969895
		Peristedion orientale	AB969896
		Scalicus amiscus	AB972262
		Scalicus engyceros	AB972261
		Satyrichthys isokawae	AB974521
	Bembridae	*Bembras japonica*	AB969899
		Bembras japonica	AB969900
	Platycephalidae	*Cociella crocodila*	AB969901
		Cociella crocodila	AB969902
		Cociella sp.	AB969903

	Hoplichthyidae	*Hoplichthys gilberti*	AB972259
		Hoplichthys gilberti	AB972260
	Hexagrammidae	*Hexagrammos agrammus*	AB969905
		Hexagrammos otakii	AB969906
		Pleurogrammus azonus	AB969907
	Cottidae	*Alcichthys elongatus*	AB972258
		Alcichthys elongatus	AB969952
		Pseudoblennius percoides	AB969908
		Stlengis misakia	AB938166
	Hemitripteridae	*Hemitripterus villosus*	AB969910
	Psychrolutidae	*Ebinania brephocephala*	AB974522
	Cyclopteridae	*Aptocyclus ventricosus*	AB969911
	Liparidae	*Careproctus* sp.	AB974682
		Liparis tanakae	AB974683
		Liparis tessellatus	AB974684
Perciformes	Acropomatidae	*Neoscombrops pacificus*	AB974574
		Doederleinia berycoides	AB974523
		Acropoma japonicum	AB974524
	Serranidae	*Caprodon schlegelii*	AB974525
		Cephalopholis aurantia	AB974527
		Cephalopholis urodeta	AB974528
		Chelidoperca hirundinacea	AB969977
		Chelidoperca pleurospilus	AB969978
		Epinephelus amblycephalus	AB972257
		Epinephelus awoara	AB972256
		Epinephelus fasciatus	AB972255
		Epinephelus lanceolatus	AB938132
		Epinephelus latifasciatus	AB972254
		Epinephelus maculatus	AB972253
		Epinephelus merra	AB972251
		Epinephelus retouti	AB972252
		Odontanthias borbonius	AB974530
		Hyporthodus octofasciatus	AB972250
		Liopropoma erythraeum	AB974534
		Plectranthias yamakawai	AB974536
		Selenanthias analis	AB974650
		Odontanthias unimaculatus	AB974532
		Niphon spinosus	AB972249
		Odontanthias rhodopeplus	AB974533
		Liopropoma aragai	AB974535
		Hyporthodus octofasciatus	AB974538
		Stereolepis doederleini	AB974539
		Diploprion bifasciatum	AB974540
		Cephalopholis urodeta	AB974529
		Caprodon schlegelii	AB974526
		Odontanthias borbonius	AB974531
		Plectranthias yamakawai	AB974537
		Aulacocephalus temmincki	AB974589
		Pseudanthias squamipinnis	AB974652
	Callanthiidae	*Grammatonotus surugaensis*	AB974651
	Opistognathidae	*Opistognathus decorus*	AB974587
	Banjosidae	*Banjos banjos*	AB974541
	Priacanthidae	*Cookeolus japonicus*	AB972247
		Heteropriacanthus cruentatus	AB972245
		Heteropriacanthus cruentatus	AB972246
		Priacanthus macracanthus	AB972244
		Priacanthus sagittarius	AB972243

Apogonidae	*Ostorhinchus franssedai*	AB972241
	Ostorhinchus notatus	AB972240
	Ostorhinchus doederleini	AB972239
Malacanthidae	*Malacanthus brevirostris*	AB972238
	Branchiostegus japonicus	AB972237
	Branchiostegus auratus	AB972236
Coryphaenidae	*Coryphaena hippurus*	AB938133
Rachycentridae	*Rachycentron canadum*	AB972181
Echeneidae	*Echeneis naucrates*	AB969989
	Phtheirichthys lineatus	AB938134
	Phtheirichthys lineatus	AB938135
	Remora remora	AB938136
	Remorina albescens	AB974542
	Echeneis naucrates	AB974595
Carangidae	*Alectis ciliaris*	AB938139
	Carangoides ferdau	AB969992
	Carangoides orthogrammus	AB938141
	Caranx ignobilis	AB938142
	Caranx melampygus	AB969991
	Caranx papuensis	AB969994
	Caranx sexfasciatus	AB969990
	Decapterus macrosoma	AB969924
	Decapterus maruadsi	AB969925
	Decapterus muroadsi	AB969927
	Decapterus tabl	AB969926
	Elagatis bipinnulata	AB969993
	Gnathanodon speciosus	AB969998
	Carangoides equula	AB974543
	Pseudocaranx dentex	AB969997
	Scomberoides lysan	AB938137
	Selene vomer	AB938140
	Seriola dumerili	AB969928
	Seriola rivoliana	AB938138
	Trachurus japonicus	AB972182
	Uraspis helvola	AB969955
	Carangoides dinema	AB969995
	Seriolina nigrofasciata	AB972178
	Naucrates ductor	AB972179
	Megalaspis cordyla	AB972223
	Carangoides caeruleopinnatus	AB974544
	Carangoides caeruleopinnatus	AB974545
	Trachinotus blochii	AB974596
Menidae	*Mene maculata*	AB974593
Leiognathidae	*Nuchequula nuchalis*	AB974546
Bramidae	*Pterycombus petersii*	AB938174
	Taractes rubescens	AB972180
	Eumegistus illustris	AB972222
Emmelichthyidae	*Emmelichthys struhsakeri*	AB972221
	Erythrocles schlegelii	AB972220
Lutjanidae	*Aphareus furca*	AB974573
	Etelis coruscans	AB969963
	Etelis radiosus	AB969964
	Lutjanus bohar	AB938144
	Lutjanus bohar	AB938145
	Lutjanus decussatus	AB938147
	Lutjanus fulvus	AB974575
	Lutjanus ophuysenii	AB972219

	Lutjanus russellii	AB969971
	Lutjanus russellii	AB969970
	Lutjanus sebae	AB974547
	Lutjanus stellatus	AB972218
	Macolor macularis	AB974548
	Paracaesio sordida	AB969973
	Paracaesio sordida	AB969972
	Pristipomoides auricilla	AB969966
	Pristipomoides sieboldii	AB969967
	Pristipomoides zonatus	AB969968
	Randallichthys filamentosus	AB974644
	Paracaesio caerulea	AB974645
	Lutjanus monostigma	AB972217
	Pristipomoides sieboldii	AB972216
	Lutjanus fulviflamma	AB972215
	Paracaesio stonei	AB974646
	Aphareus rutilans	AB974647
	Pristipomoides flavipinnis	AB974549
	Pristipomoides argyrogrammicus	AB974550
Caesionidae	*Caesio caerulaurea*	AB969996
	Caesio cuning	AB938148
	Caesio teres	AB969981
	Pterocaesio digramma	AB938149
	Pterocaesio marri	AB938150
	Pterocaesio tile	AB969982
Gerreidae	*Gerres oyena*	AB974551
	Gerres macracanthus	AB974601
Haemulidae	*Plectorhinchus cinctus*	AB969919
	Plectorhinchus lineatus	AB969920
	Plectorhinchus schotaf	AB969921
Hapalogenyidae	*Hapalogenys analis*	AB969922
	Hapalogenys kishinouyei	AB969953
	Hapalogenys nigripinnis	AB969923
	Hapalogenys analis	AB974552
Nemipteridae	*Nemipterus zysron*	AB972210
	Parascolopsis inermis	AB974553
	Parascolopsis eriomma	AB974554
	Pentapodus nagasakiensis	AB974555
	Pentapodus aureofasciatus	AB974556
Lethrinidae	*Gnathodentex aureolineatus*	AB974557
	Gymnocranius euanus	AB969985
	Gymnocranius grandoculis	AB969983
	Gymnocranius lethrinoides	AB969984
	Lethrinus genivittatus	AB938152
	Lethrinus haematopterus	AB969969
	Lethrinus microdon	AB969987
	Lethrinus nebulosus	AB969986
	Lethrinus nebulosus	AB938153
	Lethrinus olivaceus	AB969988
	Lethrinus rubrioperculatus	AB938154
	Wattsia mossambica	AB972212
	Lethrinus semicinctus	AB972234
	Lethrinus genivittatus	AB974591
Sparidae	*Acanthopagrus latus*	AB972185
	Acanthopagrus sivicolus	AB972183
	Dentex abei	AB972209
	Rhabdosargus sarba	AB972208

	Acanthopagrus sivicolus	AB972184
	Dentex tumifrons	AB972235
	Dentex hypselosomus	AB974558
Polynemidae	*Polydactylus plebeius*	AB972207
Sciaenidae	*Larimichthy crocea*	AB972205
	Pennahia argentata	AB969962
	Nibea mitsukurii	AB972233
Mullidae	*Parupeneus multifasciatus*	AB972203
	Upeneus japonicus	AB972201
	Upeneus japonicus	AB972202
	Upeneus subvittatus	AB972200
	Parupeneus pleurostigma	AB974559
	Parupeneus ciliatus	AB974581
Kyphosidae	*Kyphosus cinerascens*	AB972197
Girellidae	*Girella punctata*	AB972232
Microcanthidae	*Microcanthus strigatus*	AB972198
Chaetodontidae	*Chaetodon daedalma*	AB969959
	Chaetodon nippon	AB972195
	Forcipiger flavissimus	AB969979
	Roa modesta	AB974560
Pomacanthidae	*Apolemichthys trimaculatus*	AB972199
	Chaetodontoplus septentrionalis	AB972194
	Genicanthus bellus	AB974590
Pentacerotidae	*Evistias acutirostris*	AB972193
	Pentaceros japonicus	AB974643
Terapontidae	*Rhyncopelates oxyrhynchus*	AB972231
Kuhliidae	*Kuhlia rupestris*	AB972190
Cheilodactylidae	*Cheilodactylus quadricornis*	AB938159
	Cheilodactylus zebra	AB938160
Cepolidae	*Acanthocepola indica*	AB969935
	Acanthocepola krusensternii	AB969937
	Acanthocepola limbata	AB969936
	Owstonia grammodon	AB969934
	Owstonia tosaensis	AB972188
Embiotocidae	*Ditrema temminckii temminckii*	AB969917
	Neoditrema ransonneti	AB969916
Pomacentridae	*Abudefduf vaigiensis*	AB969974
	Chromis mirationis	AB974585
	Amphiprion clarkii	AB969975
	Chromis flavomaculata	AB969958
	Chromis flavomaculata	AB969957
	Chromis notatus notatus	AB969956
	Dascyllus trimaculatus	AB972177
	Pomacentrus taeniometopon	AB972176
Labridae	*Anampses meleagrides*	AB974608
	Bodianus leucostictus	AB974606
	Bodianus oxycephalus	AB974605
	Cheilinus lunulatus	AB972175
	Cheilio inermis	AB972173
	Coris dorsomacula	AB972172
	Coris picta	AB972171
	Halichoeres tenuispinis	AB972170
	Hologymnosus doliatus	AB972169
	Iniistius twistii	AB972166
	Labroides dimidiatus	AB972165
	Novaculops woodi	AB972164
	Oxycheilinus bimaculatus	AB972163

	Oxycheilinus bimaculatus	AB972162
	Oxycheilinus unifasciatus	AB972161
	Parajulis poecilepterus	AB972160
	Pseudolabrus sieboldi	AB972159
	Pteragogus aurigarius	AB972158
	Pteragogus aurigarius	AB972157
	Pteragogus aurigarius	AB972156
	Stethojulis interrupta terina	AB972155
	Thalassoma hardwicke	AB972154
	Iniistius pavo	AB972153
	Iniistius pavo	AB972152
	Cheilinus undulatus	AB972168
	Choerodon robustus	AB972167
	Novaculichthys taeniourus	AB974609
	Bodianus bilunulatus	AB974607
	Oxycheilinus diagrammus	AB974610
	Semicossyphus reticulatus	AB974611
	Coris aygula	AB974580
	Oxycheilinus orientalis	AB974612
	Thalassoma lutescens	AB974583
	Choerodon fasciatus	AB974613
Scaridae	*Chlorurus frontalis*	AB972151
	Chlorurus microrhinos	AB972150
	Hipposcarus longiceps	AB972149
	Scarus dimidiatus	AB972148
	Scarus globiceps	AB972147
	Scarus niger	AB972146
	Scarus prasiognathos	AB974579
	Chlorurus japanensis	AB974602
	Scarus ghobban	AB974603
	Calotomus carolinus	AB974604
	Scarus festivus	AB974614
	Chlorurus sordidus	AB974615
	Scarus forsteni	AB974616
	Chlorurus frontalis	AB974582
	Scarus rubroviolaceus	AB974618
	Scarus forsteni	AB974617
	Scarus chameleon	AB974619
	Chlorurus oedema	AB974620
	Scarus quoyi	AB974576
	Scarus fuscocaudalis	AB974577
	Scarus prasiognathos	AB974578
	Scarus schlegeli	AB974621
	Scarus psittacus	AB974622
	Scarus spinus	AB974623
	Chlorurus bowersi	AB974624
Stichaeidae	*Lumpenella longirostris*	AB974572
Pholidae	*Pholis nebulosa*	AB972144
Ephippidae	*Chaetodipterus faber*	AB969912
Pinguipedidae	*Parapercis kamoharai*	AB974625
	Parapercis pacifica	AB974626
	Parapercis tetracantha	AB974627
	Pinguipes chilensis	AB974628
	Parapercis sexfasciata	AB974629
Percophidae	*Acanthaphritis unoorum*	AB972143
Uranoscopidae	*Uranoscopus chinensis*	AB974630
	Uranoscopus japonicus	AB974631

Blenniidae	*Istiblennius edentulus*	AB969914
	Petroscirtes breviceps	AB969915
	Salarias fasciatus	AB974632
	Petroscirtes breviceps	AB974633
	Omobranchus punctatus	AB974635
	Petroscirtes breviceps	AB974634
Callionymidae	*Calliurichthys japonicus*	AB970001
Eleotridae	*Ophieleotris sp.*	AB974563
	Ophiocara porocephala	AB974564
Gobiidae	*Pterogobius virgo*	AB972141
	Cabillus sp. Domori	AB972139
	Cabillus lacertops	AB972138
	Cabillus tongarevae	AB972137
	Priolepis akihitoi	AB972136
	Priolepis borea	AB972135
	Priolepis cincta	AB972134
	Priolepis fallacincta	AB972133
	Priolepis inhaca	AB972132
	Priolepis_latifascima	AB972131
	Priolepis semidoliata	AB972130
	Priolepis sp. Tsuchi-hama	AB972129
	Bathygobius fuscus	AB972128
	Sicyopterus japonicus	AB974561
	Stenogobius sp.	AB974562
	Periophthalmus argentilineatus	AB974565
	Callogobius tanegasimae	AB974566
	Exyrias puntang	AB974567
	Psammogobius biocellatus	AB974568
	Glossogobius olivaceus	AB974636
	Yongeichthys criniger	AB974569
	Trimma macrophthalma	AB974653
	Trimma annosum	AB974654
	Trimma caesiura	AB974655
	Trimma cana	AB974656
	Trimma cana	AB974657
	Trimma emeryi	AB974658
	Trimma flavatrum	AB974659
	Trimma grammistes	AB974660
	Trimma hayashii	AB974661
	Trimma kudoi	AB974662
	Trimma marinae	AB974663
	Trimma milta	AB974664
	Trimma naudei	AB974665
	Trimma okinawae	AB974666
	Trimma rubromaculatum	AB974667
	Trimma sheppardi	AB974668
	Trimma sp.	AB974669
	Trimma taylori	AB974670
	Trimma tevegae	AB974671
	Trimma yanagitai	AB974672
	Favonigobius reichei	AB974685
	Redigobius bikolanus	AB974686
Ephippidae	*Platax orbicularis*	AB972127
Siganidae	*Siganus guttatus*	AB972126
	Siganus fuscescens	AB974570
Zanclidae	*Zanclus cornutus*	AB974571
Acanthuridae	*Acanthurus achilles*	AB972125

		Acanthurus blochii	AB972124
		Acanthurus dussumieri	AB972123
		Acanthurus lineatus	AB972122
		Acanthurus nigricauda	AB972121
		Acanthurus nigrofuscus	AB972120
		Acanthurus olivaceus	AB972119
		Ctenochaetus striatus	AB972118
		Naso hexacanthus	AB938155
		Prionurus scalprum	AB972117
		Naso maculatus	AB972116
	Gempylidae	*Ruvettus pretiosus*	AB972230
	Trichiuridae	*Evoxymetopon macrophthalmus*	AB969931
		Lepidopus caudatus	AB938176
		Tentoriceps cristatus	AB938177
		Trichiurus sp. 1	AB938178
		Trichiurus japonicus	AB972229
		Trichiurus sp. 2	AB938179
	Scombridae	*Euthynnus affinis*	AB938158
		Grammatorcynus bilineatus	AB938157
		Rastrelliger kanagurta	AB938156
		Scomber japonicus	AB972113
	Istiophoridae	*Kajikia audax*	AB972112
		Istiophorus platypterus	AB974637
	Centrolophidae	*Hyperoglyphe antarctica*	AB938180
		Hyperoglyphe japonica	AB972111
		Schedophilus velaini	AB969932
	Nomeidae	*Cubiceps baxteri*	AB938181
		Cubiceps squamiceps	AB938182
		Psenes arafurensis	AB938183
		Psenes pellucidus	AB972110
		Psenes pellucidus	AB972109
	Tetragonuridae	*Tetragonurus pacificus*	AB938184
	Caproidae	*Antigonia rubescens*	AB972106
		Antigonia capros	AB974638
		Antigonia capros	AB974639
Pleuronectiformes	Citharidae	*Citharoides macrolepidotus*	AB972105
	Paralichthyidae	*Paralichthys olivaceus*	AB972103
		Pseudorhombus oligodon	AB972096
	Pleuronectidae	*Eopsetta grigorjewi*	AB972102
		Microstomus achne	AB972100
		Platichthys stellatus	AB972099
		Pleuronectes punctatissimus	AB972098
		Pleuronichthys cornutus	AB972097
		Tanakius kitaharae	AB972095
		Hippoglossoides dubius	AB972228
		Hippoglossoides pinetorum	AB972227
		Dexistes rikuzenius	AB974640
		Poecilopsetta plinthus	AB974641
		Microstomus achne	AB972101
	Bothidae	*Engyprosopon grandisquama*	AB972226
	Soleidae	*Aseraggodes kobensis*	AB972094
		Heteromycteris japonicus	AB972093
		Pseudaesopia japonica	AB972092
		Zebrias zebrinus	AB972091
		Aesopia cornuta	AB972090
		Zebrias zebrinus	AB974674
		Pardachirus pavoninus	AB972225

	Cynoglossidae	*Cynoglossus abbreviatus*	AB972089
		Cynoglossus itinus	AB972087
		Cynoglossus interruptus	AB972086
		Cynoglossus nigropinnatus	AB972224
		Cynoglossus robustus	AB974673
Tetraodontiformes	Triacanthodidae	*Triacanthodes anomalus*	AB972085
	Balistidae	*Sufflamen fraenatum*	AB972084
		Rhinecanthus aculeatus	AB974675
	Monacanthidae	*Thamnaconus hypargyreus*	AB972082
		Thamnaconus tessellatus	AB972081
		Thamnaconus modestoides	AB972080
		Aluterus monoceros	AB974676
		Rudarius ercodes	AB974677
		Stephanolepis cirrhifer	AB974678
	Ostraciidae	*Lactoria diaphana*	AB972079
		Ostracion immaculatus	AB972078
		Tetrosomus concatenatus	AB972077
	Tetraodontidae	*Sphoeroides pachygaster*	AB972075
		Takifugu niphobles	AB972074
		Chelonodon patoca	AB972073
	Diodontidae	*Cyclichthys spilostylus*	AB972072
		Diodon liturosus	AB972071
		Diodon holocanthus	AB972070
		Cyclichthys orbicularis	AB974642
	Molidae	*Masturus lanceolatus*	AB972069
		Mola sp.	AB972068
		Ranzania laevis	AB972067
